# Identification of 13 *Spirogyra* species (Zygnemataceae) by traits of sexual reproduction induced under laboratory culture conditions

**DOI:** 10.1038/s41598-019-43454-6

**Published:** 2019-05-23

**Authors:** Tomoyuki Takano, Sumio Higuchi, Hisato Ikegaya, Ryo Matsuzaki, Masanobu Kawachi, Fumio Takahashi, Hisayoshi Nozaki

**Affiliations:** 1 Department of Biological Sciences, Graduate School of Science, The University of Tokyo, 7-3-1 Hongo, Bunkyo-ku, Tokyo 113-0033 Japan; 2 Research Group for Aquatic Plants Restoration in Lake Nojiri, Nojiriko Museum, Nojiri 287-5, Shinano-machi, Kamiminochi-gun, Nagano 389-1303 Japan; 3 Graduate School of Life Science, University of Hyogo, Harima Science Park City, Hyogo, 678-1205 Japan; 4 Center for Environmental Biology and Ecosystem Studies, National Institute for Environmental Studies, 16-2 Onogawa, Tsukuba, Ibaraki 305-8506 Japan; 5 College of Life Sciences, Ritsumeikan University, Nojihigashi 1-1-1, Kusatsu-shi, Shiga 525-8577 Japan; 6 Present Address: Department of Biology, Graduate School of Science, Kobe University, 1-1 Rokkodai, Nada-ku, Kobe 657-8501 Japan

**Keywords:** Plant reproduction, Taxonomy

## Abstract

The genus *Spirogyra* is abundant in freshwater habitats worldwide, and comprises approximately 380 species. Species assignment is often difficult because identification is based on the characteristics of sexual reproduction in wild-collected samples and spores produced in the field or laboratory culture. We developed an identification procedure based on an improved methodology for inducing sexual conjugation in laboratory-cultivated filaments. We tested the modified procedure on 52 newly established and genetically different strains collected from diverse localities in Japan. We induced conjugation or aplanospore formation under controlled laboratory conditions in 15 of the 52 strains, which allowed us to identify 13 species. Two of the thirteen species were assignable to a related but taxonomically uncertain genus, *Temnogyra*, based on the unique characteristics of sexual reproduction. Our phylogenetic analysis demonstrated that the two *Temnogyra* species are included in a large clade comprising many species of *Spirogyra*. Thus, separation of *Temnogyra* from *Spirogyra* may be untenable, much as the separation of *Sirogonium* from *Spirogyra* is not supported by molecular analyses.

## Introduction

*Spirogyra* Link (Zygnemataceae, Zygnematales) is a genus in the Class Zygnematophyceae (Conjugatophyceae), which is a component member of the Infrakingdom Streptophyta^1,2^. *Spirogyra* has long been included in high school biology curricula. The genus is widely distributed in freshwater habitats including flowing water, permanent ponds and temporary pools^3^. It is characterised by its unbranched filaments made up of elongate cylindrical cells with ribbon-like chloroplasts that are spirally arranged around the cell membranes. About 380 species have been found worldwide^4–6^. The unbranched filaments and ribbon-like chloroplasts of two other confamilial genera, *Sirogonium* Kützing and *Temnogyra* Lewis^5,7^, resemble those of *Spirogyra*. These genera are readily distinguished by differences in sexual reproduction characteristics, but their vegetative morphologies are very similar. *Spirogyra* and *Temnogyra* are especially difficult to distinguish morphologically^5,7^ (Supplementary Fig. S1). Molecular phylogenetic analyses have demonstrated that *Sirogonium* is part of a clade containing diverse species of *Spirogyra*^8–11^. However, species reliably assigned to *Temnogyra* by their morphology have not been subjected to molecular analyses.

Sexual reproduction in zygnematophycean algae, such as *Spirogyra*, involves an unusual process of “conjugation” in which aplanogametes (gametes without flagella) are transferred between filaments. In *Spirogyra*, *Sirogonium* and *Temnogyra*, two gametangia of different mating types unite to form a conjugation tube through which an aplanogamete moves by amoeboid locomotion from one (male) gametangium to a second (female). A zygospore forms after gamete transfer^4^. The traits of sexual reproduction (especially mature zygospore form) are necessary for correct assignment to species and genera^4,6,7^. Previous taxonomic studies have used wild-collected filaments containing mature zygospores^9,11^. However, the seasonality of mature zygospore formation often prevents the collection of sexually mature wild filaments^12^. Less than 10% of the species of *Spirogyra*-like algae have been subjected to reliable taxonomic and phylogenetic analyses^9,11^. Laboratory cultivation to induce sexual reproduction is a promising approach for the development of a modern taxonomic system for *Spirogyra* and its relatives.

Allen^13^ induced conjugation of three vegetative types of *Spirogyra* (Groups I-III) in culture using agar plates. She identified only a single species, *S*. *pratensis*, within Group I, based on vegetative and sexual characteristics. Hoshaw *et al*.^14^ used a similar agar plate method to induce conjugation of *Spirogyra* vegetative filaments in culture and identified three species, *S*. *singularis*, *S*. *communis* and *S*. *fragilis*. Zwirn *et al*.^15^ investigated the induction of conjugation in cultures of 95 *Spirogyra* strains under diverse experimental conditions including cultures on agar plates. Although they identified eight strains as six species of *Spirogyra*, morphological traits that are essential to correctly classify these *Spirogyra* species^4,6^ were not shown. Recently, zygospores were formed with high efficiency by incubating cultured vegetative cells on agar plates of newly modified medium in two species of *Spirogyra*^16^. The procedure has not been tested on other species in the Zygnemataceae. In this study, we established clonal cultures of *Spirogyra*-like algae from many samples collected in diverse localities around Japan. Zygospores or aplanospores were induced in 13 species using the newly-modified method, which was based on the procedures of Allen^13^ and Ikegaya *et al*.^16^.

## Methods

### Sample collection and isolation

Samples containing filaments of *Spirogyra*-like algae were collected from ponds or paddy fields in Japan (Supplementary Table S1). Clones were established from fragmented filaments using a pipette-washing procedure^17^. The clones were grown in 100 mL of *Closterium* (C) medium^18^ in glass vessels (50 mm × 95 mm) held at 20 °C under a 14:10 hour light/dark cycle. We established cultures of 122 new strains of *Spirogyra*-like algae from 26 localities in Japan (Supplementary Table S1).

### Induction of conjugation

The 122 isolated strains were classifiable into 52 *rbcL*-types by differences in *rbcL* sequences; thus, we selected 52 strains with different *rbcL* sequences for further study (JPS001–JPS052; Supplementary Table S1). We attempted to induce conjugation in these strains.

In order to induce conjugation of *Spirogyra*, Allen^13^ and Ikegaya *et al*.^16^ incubated vegetative filaments on agar plates under 70–90 μmol photons m^−2^ s^−1^ illumination (500 ft-c^13^ = *ca*. 70 μmol photons m^−2^ s^−1^) on a 16:8 or 12:12 hour light/dark cycle, respectively. In this study, we used the agar plates of the medium of Ikegaya *et al*.^16^ and increased the light intensity; light intensity is reportedly important for the induction of conjugation in *Spirogyra*^13,19^.

Following the procedures of Ikegaya *et al*.^16^, we suspended agar powder (Wako, Osaka, Japan) in Artificial Pond Water medium (APW: 0.1 mM KCl, 0.1 mM CaCl_2_, 1 mM NaCl with 1 mM HEPES-Na buffer; pH 7.0) to make up 1% (w/v) agar plates in Petri dishes (90 mm × 15 mm). Actively growing filaments (*ca*. 100) in each culture were rinsed with liquid APW for 5 min and transferred onto agar plates, which were then sealed with surgical tape. The plates were incubated for *ca*. 2 weeks at 20 °C under 120 μmol photons m^−2^ s^−1^ illumination (cool-white fluorescent lamps, FL40SW; NEC Lighting, Tokyo, Japan) on a 14:10 hour light/dark cycle. Conjugation was observable after this time period when sexual reproduction had been successfully induced.

### Light microscopy

Microscopic observations were made with a BX53 microscope (Olympus, Tokyo, Japan) equipped with Nomarski differential interference optics.

### DNA sequencing

DNA extraction was performed following previously described procedures^20,21^. Cells were shaken with ceramic beads in chloroform and cetrimonium bromide using a ball mill (Mixer Mill MM 300; Retsch, Haan, Germany). DNA was extracted with an Illustra^™^ blood genomic Prep Mini Spin Kit (GE Healthcare UK, Little Chalfont, UK). RuBisCO Large subunit (*rbcL*) genes and ATP synthase beta subunit (*atpB*) were amplified by polymerase chain reaction (PCR) using previously designed primers^10,22,23^ (Supplementary Tables S2, S3). PCR products were purified with an Illustra^™^ GFX PCR DNA and Gel Band Purification Kit (GE Healthcare UK). Purified PCR products were sequenced directly using an ABI PRISM 3100 s Genetic Analyzer (Applied Biosystems, Foster City, CA, USA) with a BigDye Terminator Cycle Sequencing Ready Reaction Kit (ver. 3.1; Applied Biosystems).

### Phylogenetic analyses

We analysed 138 ingroup (Zygnematophyceae) and five outgroup (*Coleochaete*) operational taxonomic units (OTUs) of different *rbcL* sequences (Supplementary Tables S1, S4). The 138 ingroup OTUs included our 52 *rbcL-*types from Japan (Supplementary Tables S1, S4). The 1,332 base pairs of the *rbcL* gene sequences from the 143 OTUs were aligned using Clustal X software^24^. In addition, 1206 base pairs of the *atpB* gene sequences from the same 143 OTUs were aligned by Clustal X. The combined data set from *rbcL* and *atpB* genes [available from TreeBASE (https://www.treebase.org/treebase-web/home.html); study ID S24057] was subjected to maximum likelihood (ML) and Bayesian inference (BI) analyses. Then, ML analysis with 1,000 bootstrap replications^25^ was performed with RAxML v. 8.0.0 software^26^ using the GTR + CAT + I model and partitioning the dataset into first, second and third codon. BI analysis was performed with MrBayes v. 3.2.6 software^27^, as previously described^28^ (1,000,000 generations of Markov chain Monte Carlo iterations; the first 25% of the iterations were discarded as burn-in). The substitution models for each partition of BI were GTR + I + G (first and third codon positions) and K2 + I + G (second codon position), which were selected with MEGA 7.0.21 software^29^.

## Results

### Sexual reproduction

**Figure 1:**
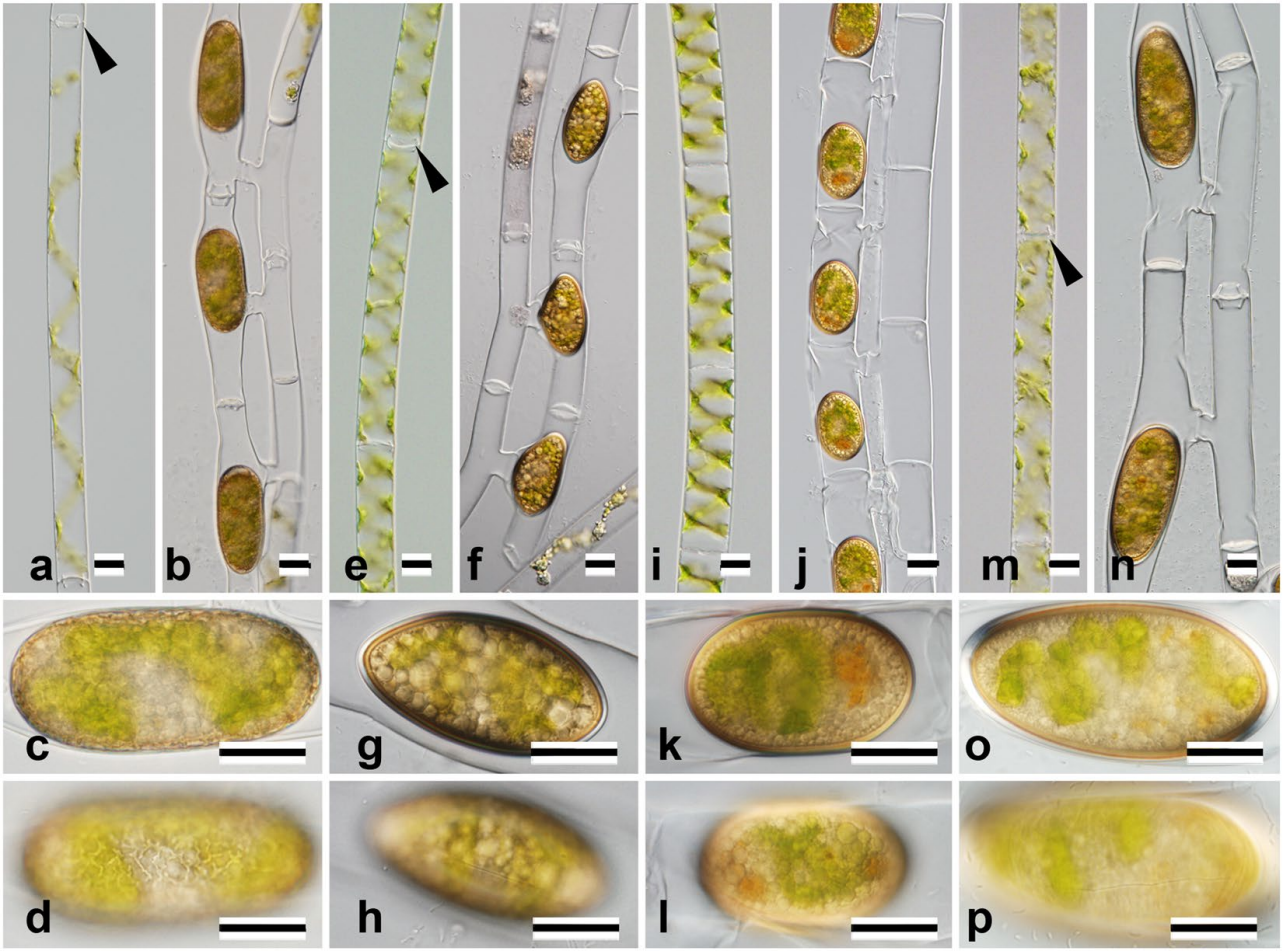
Nomarski interference micrographs of four species of *Spirogyra* belonging to Clade I (Fig. 5a, Table 1). Scale bars = 20 μm. (**a**–**d**) *S*. *dentireticulata* chiA101 (JPS003). (**e**–**h**) *S*. *hopeiensis* biw0302 (JPS004). (**i**–**l**) *S*. *longata* chiA305 (JPS005). (**m**–**p**) *S*. *semiornata* chiA304 (JPS014). (**a**,**e**,**i**,**m**) Vegetative cells. Arrowheads indicate replicate walls. **(b**,**f**,**j**,**n)** Formation of zygospores. (**c**,**g**,**k**,**o**) Optical section of zygospore. (**d**,**h**,**l**,**p**) Surface view of zygospore.

**Figure 2:**
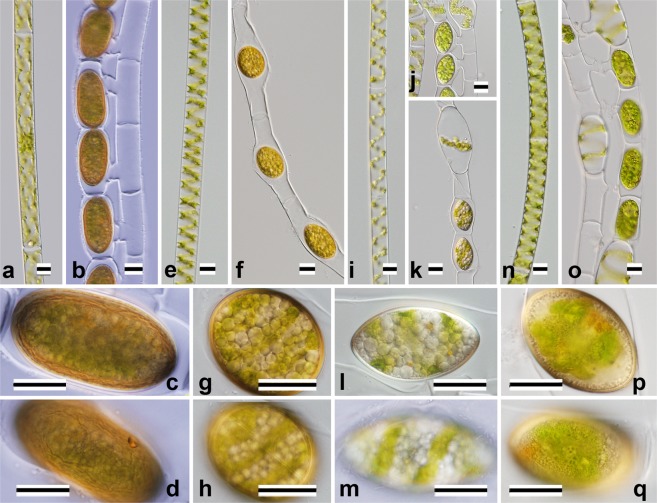
Nomarski interference micrographs of four species of *Spirogyra* belonging to Clades III–V (Fig. 5a, Table 1). Scale bars = 20 μm. (**a**–**d**) *S*. *chungkingensis* Uki1 (JPS001). (**e**–**h**) *S*. *mirabilis* shi0305 (JPS010). (**i**–**m**) *S*. *chenii* chiA307 (JPS011). (**n**–**q**) *S*. *varians* chi0102 (JPS015). (**a,e**,**i**,**n**) Vegetative cells. (**b**,**f**,**j**,**o**) Formation of zygospores or aplanospores. (**c**,**g**,**l**,**p**) Optical section of zygospore or aplanospores. (**d**,**h**,**m**,**q**) Surface view of zygospore or aplanospores.

**Figure 3:**
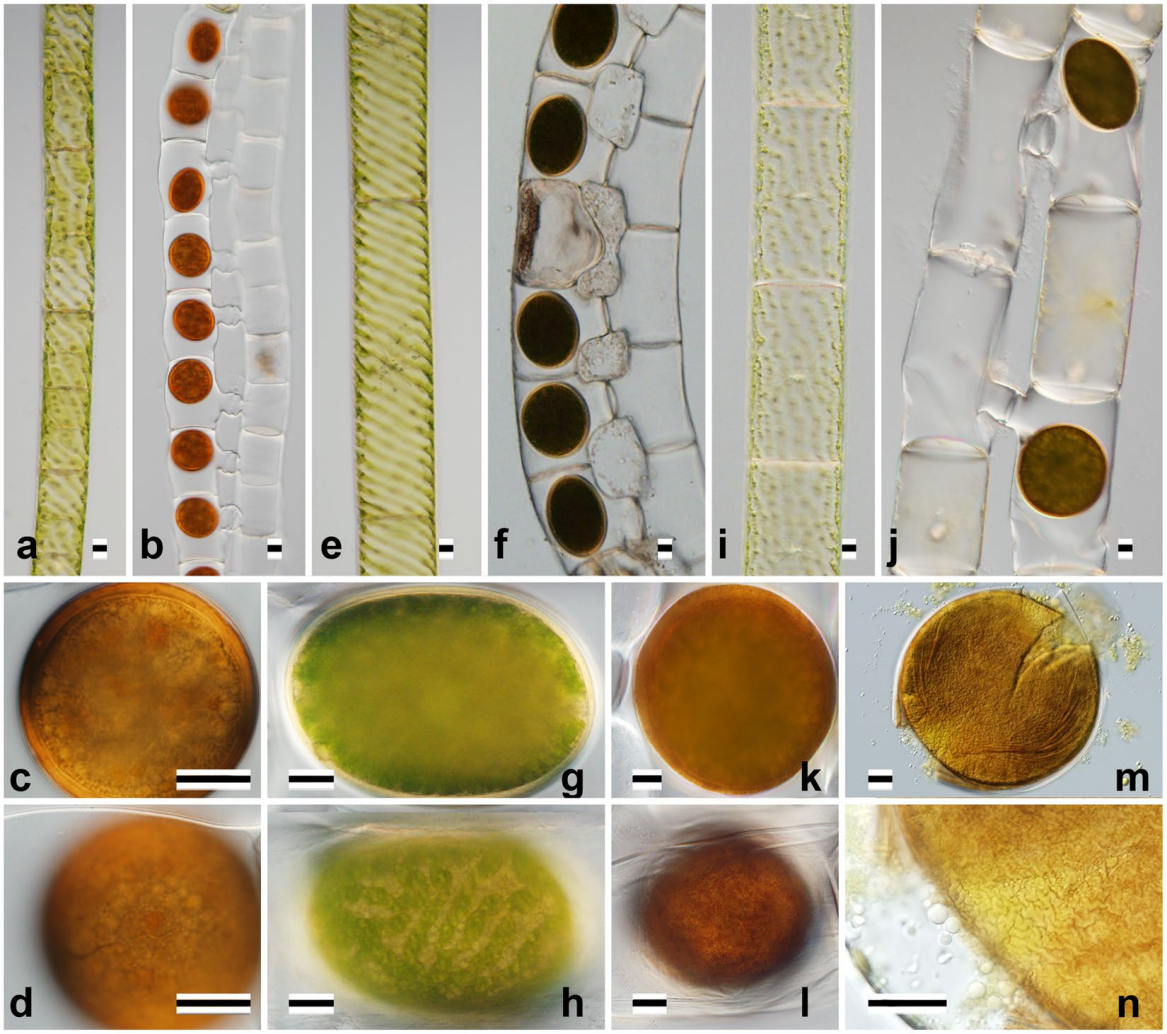
Nomarski interference micrographs of three species of *Spirogyra* belonging to Clade II, VII and having compressed zygospores (Fig. 5a, Table 1). Scale bars = 20 μm. (**a**–**d**) *S*. *majuscula* nag301 (JPS008). (**e**–**h**) *S*. *minuticrassoidea* nag101 (JPS009). (**i**–**n**) *S*. *pseudomaxima* chi0305 (JPS012). (**a**,**e**,**i**) Vegetative cells. (**b**,**f**,**j**) Formation of zygospores. (**c**,**g**,**k**) Optical section of zygospore. (**d**,**h**,**l**) Surface view of zygospore. (**m**,**n**) Crushed zygospore.

**Figure 4:**
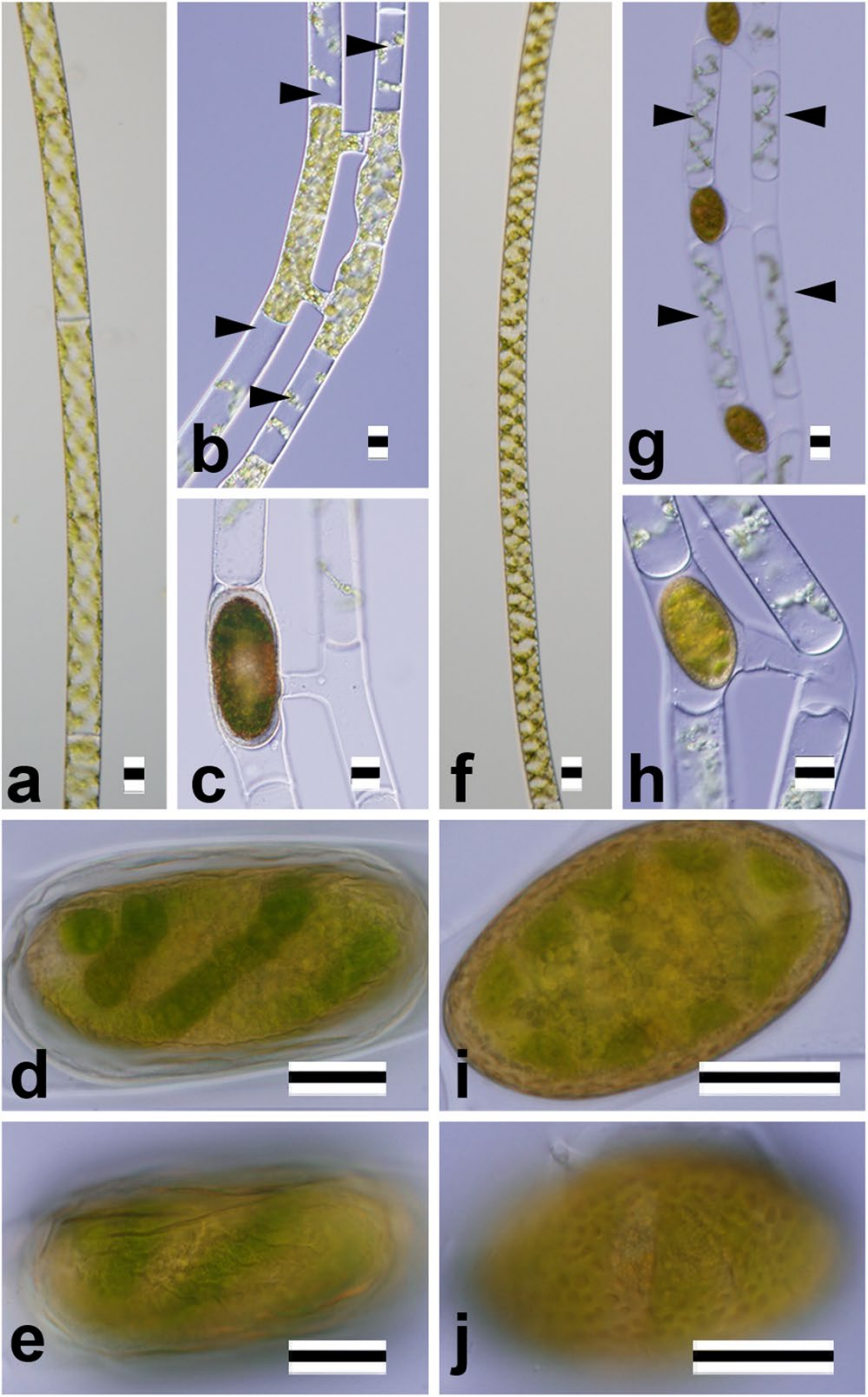
Nomarski interference micrographs of two *Spirogyra* species assignable to “*Temnogyra*” (Table 1, Supplementary Fig. S1). Scale bars = 20 μm. (**a**–**e**) *S*. *corrugata* A2F (JPS002). (**f**–**j**) *S*. *punctata* Tpx8 (JPS013). (**a**,**f**) Vegetative cells. (**b**,**c**,**g**,**h**) Formation of zygospores showing sterile cells adjoining gametangia (arrowheads in **b**,**g**). (**d**,**i**) Optical section of zygospore. (**e**,**j**) Surface view of zygospore.

Among the 52 strains with different *rbcL* sequences (Supplementary Table S1), we induced formation of zygospores or aplanospores in 15 (Figs 1–4) that were assigned to 13 species (Table 1).

**Table 1:** Comparison of the morphological characteristics of 13 *Spirogyra* species in this study.

Species	strains (*rbcL*-type*)	Width	Length	End wall	Chloroplasts per cell	Turns per cell	Vegetative cells type	Conjugation type	Gametangia shape	Zygospore	Spore membrane
*S*. *chungkingensis*	Uki1 [=NIES**-4302] (JPS001)	24–27	150–300	Plane	3	4–5	V2	C3	inflated	Z1, 33–35 × 55–83 μm	two-layered, outer brownish, wrinkled; inner brown, irregularly reticulate
*S*. *corrugata*	A2F [=NIES-4303] (JPS002)	28–34	200–600	Plane	2–3	3–5	V2	C5	inflated	Z1, 50–54 × 97–111 μm	yellow-brown, two-layered, outer thin, wrinkled, inner thick, reticulate
*S*. *dentireticulata*	chiA101 [=NIES-4304] (JPS003)	20–24	200–340	Replicate	1	4–5	V3	C3	slightly enlarged	Z1, 30–34 × 70–76 μm	yellow-brown, reticulate with coarse ridges
*S*. *hopeiensis*	biw0302 [=NIES-4305] (JPS004)	24–28	110–260	Replicate	1	5–6	V3	C4	inflated mostly on the inner sides	Z2, 30–34 × 59–62 μm	yellow, smooth
*S*. *longata*	chiA305 [=NIES-4306] (JPS005) kit0201 [=NIES-4307] (JPS006)	26–32	110–260	Plane	1	3–6	V1	C3	cylindrical	Z1, 28–32 × 48–74 μm	yellow, smooth
*S*. *majuscula*	chi0202 [=NIES-4308] (JPS007) nag301 [=NIES-4309] (JPS008)	70–80	110–300	Plane	6	1–2	V2	C3	inflated on the outer side	Z3, 58–78 × 58–78 × 43–55 μm	yellow-brown, smooth
*S*. *minuticrassoidea*	nag101 [=NIES-4310] (JPS009)	95–112	295–515	Plane	6	2.5–3	V2	C3	cylindrical	Z3 (compressed ellipsoid), 100–107 × 125–144 × 68–90 μm	yellow-brown, smooth
*S*. *mirabilis*	shi0305 [=NIES-4311] (JPS010)	20–26	105–270	Plane	1	6–8	V1	C1	inflated on both sides	Z1, 23–32 × 41–53 μm	yellow-brown, smooth
*S*. *chenii*	chiA307 [=NIES-4312] (JPS011)	17–22	160–270	Plane	1	3–5	V1	C2, C4	inflated on both sides	Z2, 24–27 × 42–52 μm	yellow, smooth
*S*. *pseudomaxima*	chi0305 [=NIES-4313] (JPS012)	135–140	190–430	Plane	7–8	1–1.5	V2	C3	cylindrical	Z3, 112–124 × 112–124 × 90–95 μm	brown, reticulate
*S*. *punctata*	Tpx8 [=NIES-4314] (JPS013)	26–30	150–300	Plane	1	5–7	V1	C5	inflated	Z2, 31–34 × 54–65 μm	yellow-brown, punctate
*S*. *semiornata*	chiA304 [=NIES-4315] (JPS014)	26–32	250–380	Replicate	1	5–6	V3	C3	cylindrical, sometimes slightly enlarged	Z1, 35–42 × 78–107 μm	yellow-brown, smooth
*S*. *varians*	chi0102 [=NIES-4316] (JPS015)	28–33	125–200	Plane	1	4–8	V1	C3	inflated toward center only	Z2, 30–34 × 50–53 μm	yellow-brown, smooth

Vegetative cell type, conjugation type and zygospore/aplanospore type are detailed in Supplementary Figs S2–S4. For details of the 13 species, see Supplementary Note S1. ^*^For strain examined in each *rbcL*-type, see Supplementary Table 1.

**Microbial Culture Collection at the National Institute for Environmental Studies^36^ (http://mcc.nies.go.jp/index_en.html).

Twelve species formed zygospores and one species formed aplanospores. Nine of the twelve species that formed zygospores developed conjugation tubes from both male and female gametangia. The other three of the twelve that formed zygospores produced conjugation tubes from only the male gametangia; two of these three underwent unequal division of the mother gametangial cell to form one small and one large daughter cell, which developed into sterile and gametangial cells, respectively. We classified the observed modes of zygospore and aplanospore formation into five categories: Types C1–C5 (Supplementary Fig. S2).

Type C1: formation of aplanospores.

Only *Spirogyra mirabilis* formed aplanospores (Fig. 3f) and its aplanosporangia were inflated. Although Transeau^4^ reported that *S*. *mirabilis* originating from North America formed both aplanospores and ladder-like conjugation tubes (Type C3), we did not observe conjugation in cultured material from Japan.

Type C2: lateral conjugation (conjugation between adjoining cells in a single filament).

Only *S*. *chenii* (Fig. 2k) had lateral conjugation.

Type C3: ladder-like conjugation (conjugation between cells in two adjoining filaments), conjugation tubes formed by both gametangia.

Nine species belonged to this category (*S*. *dentireticulata* [Fig. 1b]: *S*. *longata* [Fig. 1j], *S*. *semiornata* [Fig. 1n], *S*. *chungkingensis* [Fig. 2b], *S*. *varians* [Fig. 2o], *S*. *majuscula* [Fig. 3b], *S*. *minuticrassoidea* [Fig. 3f], and *S*. *pseudomaxima* [Fig. 3j]).

Type C4: ladder-like conjugation; conjugation tubes often formed by a papilla from the male gametangium, lacking sterile cells adjacent to gametangia.

*S*. *hopeiensis* (Fig. 1f) and *S*. *chenii* (Fig. 2j) belonged to this category.

Type C5 (*Temnogyra*-type): ladder-like conjugation; conjugation tubes often formed by a papilla from the male gametangium, sterile cells adjacent to gametangia.

*S*. *corrugata* (Fig. 4b) and *S*. *punctata* (Fig. 4g) were members of this category.

### Mature zygospores and aplanospores

We recognised three types of zygospores or aplanospores (Types Z1–Z3; Supplementary Fig. S3). In the first type, the zygospores and aplanospores were ovoid in shape (resembling a watermelon)^4^, and the mesospores were single- or double-layered (Type Z1). In the second type, the zygospore was ellipsoidal (resembling an American football)^4^ and the mesospores were single-layered (Type Z2). In the third type, the zygospore was lenticular (a compressed spheroid) and the mesospores were single- or double-layered (Type Z3).

Type Z1: ovoid zygospore/aplanospore.

Six of the species that we studied belonged to this category. Three species (*S*. *longata* [Fig. 1k,l], *S*. *semiornata* [Fig. 1o,p], and *S*. *mirabilis* [Fig. 2g,h]) had smooth, single-layered mesospores, but *S*. *dentireticulata* (Fig. 1c,d) had reticulate, single-layered mesospores. *S*. *chungkingensis* (Fig. 2c,d) and *S*. *corrugata* (Fig. 4d,e) had ornamented, double-layered mesospores.

Type Z2: ellipsoid zygospore.

Four species belonged to this category. Three species (*S*. *hopeiensis* [Fig. 1g,h], *S*. *chenii* [Fig. 2l,m], and *S*. *varians* [Fig. 2p,q]) had smooth, single-layered mesospores; *S*. *punctata* (Fig. 4i,j) had punctate, single-layered mesospores.

Type Z3: lenticular zygospore.

This zygospore type occurred in three species. Two species (*S*. *majuscula* [Fig. 3e,g] and *S*. *minuticrassoidea* [Fig. 3g,h]) had smooth, single-layered mesospores; *S*. *pseudomaxima* (Fig. 3k,l) had an ornamented double-layered mesospore.

### Vegetative morphology

Vegetative cells of the 52 strains were classified into three categories (Types V1–V3; Supplementary Fig. S4 and Table S5), based on differences in the transverse cell walls and numbers of chloroplasts.

Type V1: plane transverse wal l and single chloroplast.

Cells of this type were relatively narrow (10–47 μm wide). Among the 13 species recognised morphologically (Table 1), five were members of this category (*S*. *longata* [Fig. 1i], *S*. *mirabilis* [Fig. 2e], *S*. *chenii* [Fig. 2i], *S*. *varians* [Fig. 2n], and *S*. *punctata* [Fig. 4f]).

Type V2: plane transverse wall and multiple chloroplasts.

Five of the thirteen species (Table 1) were members of this category; three of the five had broad vegetative cells (*ca*. 70–140 μm wide): *S*. *majuscula* (Fig. 3a), *S*. *minuticrassoidea* (Fig. 3e) and *S*. *pseudomaxima* (Fig. 3i). Two of the five had narrow cells (*ca*. 30 μm wide): *S*. *chungkingensis* (Fig. 2a) and *S*. *corrugata* (Fig. 4a).

Type V3: replicate transverse wall and single chloroplast.

Cells of this type were relatively narrow (8–32 μm wide). Three of the thirteen species (Table 1) belonged to this category (*S*. *dentireticulata* [Fig. 1a], *S*. *hopeiensis* [Fig. 1e], and *S*. *semiornata* [Fig. 1m]).

### Molecular phylogeny

**Figure 5:**
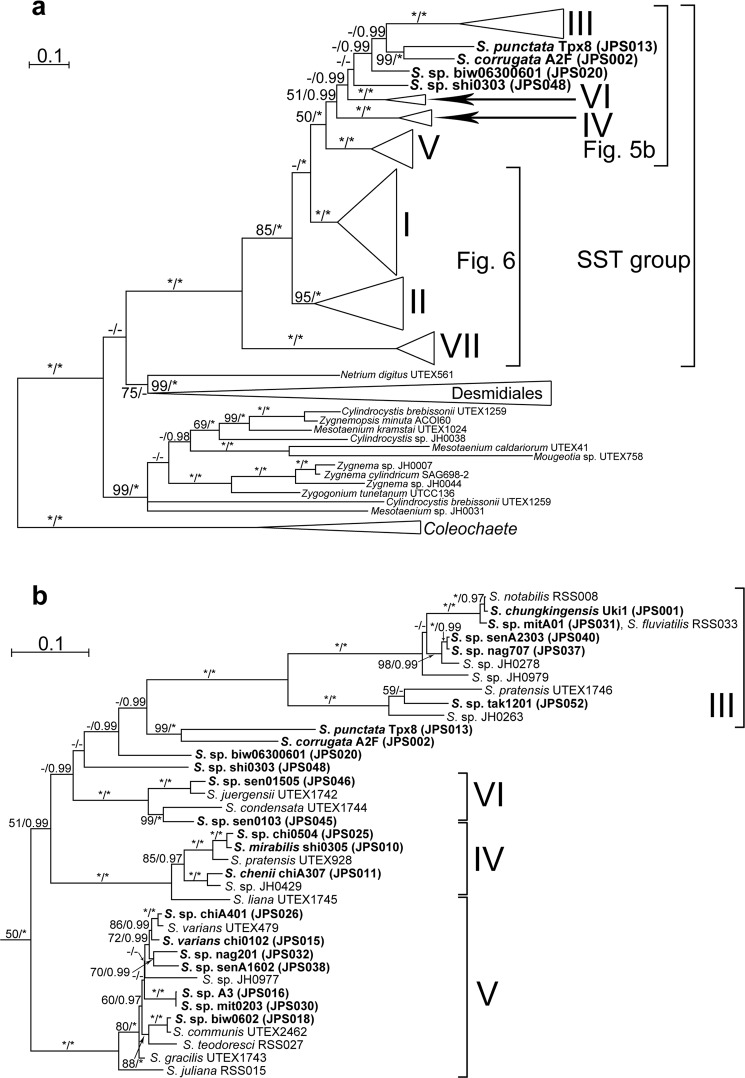
Maximum likelihood (ML) tree based on the combined data set from *rbcL* and *atpB* genes of 138 operational taxonomic units (OTUs) (zygnematophycean algae) and five outgroup OTUs (*Coleochaete*) (Supplementary Tables S1, S4) using RAxML^26^. Branch lengths are proportional to the evolutionary distances indicated by scale bar above the tree or subtree in each figure. Numbers at left and right sides above branches are bootstrap values (50% or more) by ML analysis and posterior probabilities (0.90 or more) by Bayesian inference (BI), respectively. Asterisk represents 100% bootstrap values by ML method or 1.00 posterior probability by BI. OTU designations in boldface represent 52 *rbcL*-types from Japan (Supplementary Table S1). Note that OTUs of I–VII correspond to those of Clades I–VII of Stancheva *et al*.^11^ and “*S*. sp.” may belong to *Temnogyra* or *Sirogonium* because of lack of sexual reproduction characteristics. (**a**) Overview of the ML tree based on *rbcL* and *atpB* concatenated dataset. Details of “Desmidiales” are shown in Supplementary Fig. S5. (**b**) Details of part of the ML tree (**a**) showing Clades III–VI.

**Figure 6:**
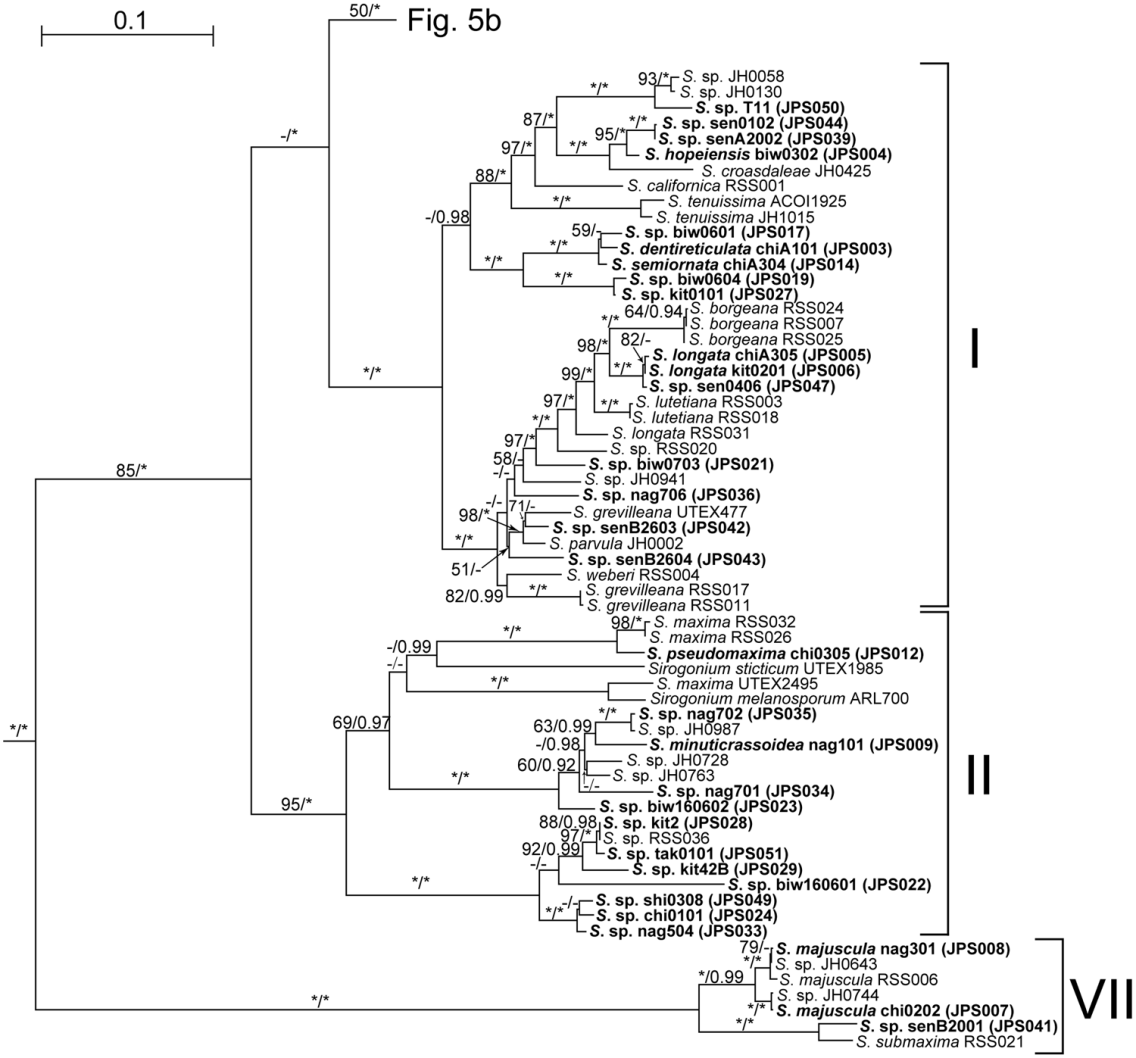
Details of part of the ML tree based on the combined data set from *rbcL* and *atpB* genes (Fig. 5a) showing a section corresponding to Clades I, II and VII of Stancheva *et al*.^11^. For details of the explanation of the tree, see Fig. 5.

All of the OTUs of *Spirogyra* (including “*Temnogyra*”) and *Sirogonium* that we analysed belonged to a large monophyletic group (SST group) with high support values (100% bootstrap values [BV] in ML analysis; 1.00 posterior probability [PP] in BI analysis). Outside this monophyletic group, two robust clades corresponding to Zygnematales (except for *Netrium digitus*) and Desmidiales were resolved with high support values (99% BV and 1.00 PP). The former clade contained filamentous algae (*Zygnema*, *Zygnemopsis*, *Zygogonium* and *Mougeotia*) and unicellular algae (*Cylindrocystis* and *Mesotaenium*) (Fig. 5a). *Netrium digitus* was sister to Desmidiales with low support values (only 75% BV) as in previous studies^8,10,30^. Within SST group, seven clades corresponding to Clades I–VII in Stancheva *et al*.^11^ were robustly resolved (with ≥95% BV and 1.00 PP) (Fig. 5a). Each of these seven clades (Clades I–VII) included OTUs of new Japanese strains that we established in this study (Figs 5b and 6); however, four of the Japanese OTUs or *rbcL* types that we identified were positioned outside the clades resolved in this study (Fig. 5a,b). These four OTUs included two species (*S*. *corrugata* A2F (JPS002) and *S*. *punctata* Tpx8 (JPS013)) that were assignable to *Temnogyra* based on the sexual reproduction characteristics that we observed (Fig. 4). These two “*Temnogyra*” species were robustly monophyletic (with 99% BV and 1.00 PP).

Clade I contained all species with replicate transverse walls (type V3; e.g. *S*. *dentireticulata* chiA101 [JPS003], *S*. *hopeiensis* biw0302 [JPS004], and *S*. *semiornata* chiA304 [JPS014]), and several strains with plane transverse walls (type V1; e.g. *S*. *longata* chiA305 [JPS005] and kit0201 [JPS006]) (Figs 1 and 6). Vegetative cells of all of the identified species in this clade were relatively narrow (20–32 μm wide) with one chloroplast per cell^4,11^. Sexual reproduction and zygospore types were diverse (Types C3, C5, Z1 and Z2; Table 1).

Clade II (Fig. 6) included some species with lenticular zygospores (type Z3) (*S*. *minuticrassoidea* nag101 [JPS009] [Fig. 3g,h] and *S*. *pseudomaxima* chi0305 [JPS012] [Fig. 3k–n]). Sexual reproduction was type C3 in all species within this clade. The identified species in this clade had relatively broad vegetative cells (95–150 μm diameter^11^; type V2).

Clade III (Fig. 5b) contained *S*. *chungkingensis* Uki1 (JPS001) (Fig. 2a–d), *S*. *fluviatilis* and *S*. *notabili*s. These species had multiple chloroplasts in the vegetative cells (V2), and complex, multi-layered, ornamented mesospores in the zygospores (Fig. 2)^4^.

Clade IV (Fig. 5b) contained *S*. *mirabilis* shi0305 (JPS010) (Fig. 2e–h), *S*. *chenii* chiA307 (JPS011) (Fig. 2i–m) *S*. *pratensis* UTEX928 and *S*. *liana* UTEX1745. All of these species had smooth mesospores^4^.

Clade V (Fig. 5b) contained *S*. *varians* chi0102 (JPS015) (Fig. 2n–q) and *S*. *varians* strains from California (RSS013), India (UTEX479). The vegetative cells in all of the identified members in this clade (*S*. *varians* chi0102 [JPS015], *S*. *communis* UTEX2462, *S*. *teodoresci* RSS027, *S*. *juliana* RSS015, and *S*. *gracilis* UTEX1743) contained one or two chloroplasts (Type V1); the mesospores were smooth or ornamented (Fig. 2)^4,11^.

Clade VI (Fig. 5b) contained only four strains, including the two unidentified Japanese strains JPS045 and JPS046.

Clade VII (Fig. 6) contained *S*. *majuscula* chi0202 (JPS007) and nag301 (JPS008) (Fig. 3c,d) and *S*. *submaxima* RSS021^11^. These species had lenticular zygospores (type Z3) and their vegetative cells were relatively broad (60–105 μm diameter^11^; type V2). Sexual reproduction was type C3 in all species within this clade.

## Discussion

The 15 strains in which zygospores or aplanospores were induced were clearly assignable to 13 species (Table 1). Two of the thirteen species (*S*. *corrugata* and *S*. *punctata*) were assignable to *Temnogyra* according to their sexual reproduction characteristics (Fig. 4). These two species formed a robust clade separated from *Sirogonium* species within a large monophyletic group (SST group) that included all of the *Spirogyra* OTUs examined in our phylogenetic analysis (Fig. 5a). Therefore, the two “*Temnogyra*” species should be re-assigned to the genus *Spirogyra*. However, information on the type species of *Temnogyra*, *T*. *collinsii* I. F. Lewis^31^, was not available during the present study. Further studies that include *T*. *collinsii* will be needed to clarify the taxonomic status of the genus *Temnogyra*.

UTEX 1745 is labelled “*Spirogyra liana*”^32^ in the Texas culture collection; it has been the subject of previous phylogenetic analyses, and we included it in our study (Fig. 5b)^11^. *S*. *liana* has been recognised as a member of “*Temnogyra*” based on its conjugation characteristics^7^. However, there is no information on the sexual reproduction traits of UTEX 1745. This strain was phylogenetically separated from the two species assigned to *Temnogyra* (*S*. *corrugata* and *S*. *punctata*) based on their peculiar sexual reproduction characteristics (Fig. 4).

We obtained molecular information on eight *Spirogyra* species for the first time. Among these, there were three rare species: the Japanese endemic *S*. *minuticrassoidea*, and two species that had not previously been recorded in Japan, *S*. *pseudomaxima* and *S*. *dentireticulata*. These species probably seldom reproduce sexually in the wild. Hence, our cultivation procedure for inducing conjugation in vegetative cells proved useful for the identification of *Spirogyra* species that seldom, or rarely, sexually reproduced in nature. It is likely that more cryptic species will be revealed by inducing sexual reproduction in a range of established culture strains.

Among 52 genetically different strains, we were able to identify 15 that were assignable to 13 species. We were not able to obtain adequate information on conjugation in the remaining 37 strains, which were consequently unidentifiable at the species level. The difficulty of inducting sexual reproduction may have been a product of the procedure that we used. We attempted to induce conjugation using single clonal cultures to identify homothallic sexuality. Although heterothallic sexuality has not been demonstrated previously in *Spirogyra* or *Sirogonium*^11,33,34^, it cannot be ruled out for the culture strains that we were unable to assign to species. The procedures for induction should therefore be modified and improved so that the taxonomic status of strains such as these can be resolved.

*Spirogyra*-like algae are diverse with approximately 380 species widely distributed all over the world^4–6,9,11^. However, only 13 species of these algae were correctly identified in at species level using cultured materials originating only from Japan (Table 1). Thus, further taxonomic studies are needed based on more culture strains of *Spirogyra*-like algae from various localities of the world in order to construct more reliable and through taxonomic systems of *Spirogyra* and its related genera. However, some problems are recognized in species with wide distribution. *S*. *varians* UTEX479 collected in England and *S*. *varians* chi0102 (JPS015) from Japan are closely related (Fig. 5b). In contrast, *S*. *longata* RSS031 from California and *S*. *longata* chiA305 (JPS005) and kit0201 (JPS006) collected in Japan (Supplementary Table S1) are phylogenetically separated (Fig. 6). Similarly, *S*. *majuscula* RSS006 from California and *S*. *majuscula* chi0202 (JPS007) (Supplementary Table S1) from Japan are separated, as well as *S*. *pratensis* UTEX928 from USA and *S*. *pratensis* UTEX1746 from India as discussed in the previous studies (Figs 5b and 6)^8,11^. Therefore, morphological species concept based on only light microscopic characteristics of vegetative and reproductive phases may not delineate natural or monophyletic species as previously suggested^35^. Although it is necessary to induce and examine sexual reproduction characteristics of more worldwide culture strains to progress taxonomic studies of *Spirogyra*-like algae, more detailed observations using scanning and transmission electron microscopes combined with molecular phylogeny are need to recognize actual species of these algae.

## Supplementary information

Supplementary Information for: Identification of 13 Spirogyra species (Zygnemataceae) by traits of sexual reproduction induced under laboratory culture conditions

## Data Availability

All the other data generated and analysed during this study are included in this published article and its Supplementary Information.
