# Supplementary material for: Identification of 13 Spirogyra species (Zygnemataceae) by traits of sexual reproduction induced under laboratory culture conditions
Source: Sci Rep. 2019 May 23;9:7458. doi: 10.1038/s41598-019-43454-6 (PMC6533312; doi:10.1038/s41598-019-43454-6)
Supplement: Supplementary file 1 — Supplementary Information for: Identification of 13 Spirogyra species (Zygnemataceae) by traits of sexual reproduction induced under laboratory culture conditions [file 41598_2019_43454_MOESM1_ESM.pdf]

Supplementary Information for:

Identification of 13 *Spirogyra* species (Zygnemataceae) by traits of sexual reproduction induced under laboratory culture conditions

Tomoyuki Takano, Sumio Higuchi, Hisato Ikegaya, Ryo Matsuzaki, Masanobu Kawachi, Fumio Takahashi and Hisayoshi Nozaki

Contents:

Supplementary Tables

Supplementary Table S1. List of localities of samples from which 122 new strains of *Spirogyra* (*S.*) were collected.

Supplementary Table S2. List of primers used for sequencing *rbcL* genes.

Supplementary Table S3. List of primers used for sequencing *atpB* genes.

Supplementary Table S4. List of additional *rbcL* and *atpB* gene sequences to Table S1, included in the present phylogenetic analysis.

Supplementary Table S5. Information of vegetative cells about 52 strains in this study.

Supplementary Figures

Supplementary Figure S1. Comparison of conjugation processes in *Spirogyra* and *Temnogyra*.

Supplementary Figure S2. Diagrams of five types of formation of zygospores or aplanospore observed in this study.

Supplementary Figure S3. Diagrams of three types of zygospores or aplanospores observed in this study.

Supplementary Figure S4. Diagrams of three types of vegetative cells observed in this study.

Supplementary Figure S5. Details of "Desmidiales" in ML tree (Fig. 5a).

Supplementary Notes

Supplementary Note S1. Taxonomic accounts.

References for Supplementary Information

Supplementary Tables

Supplementary Table S1. List of localities of samples from which 122 new strains of *Spirogyra* (*S.*) were collected.

Locality and habitat	Water temperature (℃)	pH	Strain*	Species and *rbcL*-type**	New *rbc*L genes used for present phylogenetic analysis	New *atpB* genes used for present phylogenetic analysis
A pond in Toneri park, Tokyo (35°47'52.1"N 139°46'23.0"E) (29 November 2013)	11	7.4	T8, T15	*S.* sp.*** JPS037		
			T9	*S.* sp. JPS050		
			T11	*S.* sp. JPS050	MK558113	MK558151
A pond in Ukima park, Toyko (35°47'33.5"N 139°41'31.7"E) (11 December 2013)	10	8.2	Uki1 [=NIES-4302]	*S. chungkingensis* Jao JPS001	MK558136	MK558167
			Uki2	*S.* sp. JPS001		
			Uki5, Uki9, Uki11	*S.* sp. JPS050		
A pond in Mitsugi park, Tokyo (35°46'21.3"N 139°41'37.6"E) (14 December 2013)	10	7.1	mit0203	*S.* sp. JPS030	MK558104	MK558176
			mitA01	*S.* sp. JPS031	MK558135	MK558186
			mitA02	*S.* sp. JPS031		
			mitA06, mitA07	*S.* sp. JPS030		
			mitA04, mitA05	*S.* sp. JPS001		
A pond in Arakaza Sizen park, Tokyo (35°44'24.5"N 139°47'06.8"E) (25 December 2013)	6	7.6	A3	*S.* sp. JPS016	MK558103	MK558175
			A6	*S.* sp. JPS016		
			A4	*S.* sp. JPS050		
			A5	*S.* sp. JPS050		
			A2F [=NIES-4303]	*S. corrugata* Transeau JPS002	MK558126	MK558170
			Tpx8 [=NIES-4314]	*S. punctata* Cleve JPS013	MK558127	MK558169
Paddy fields in Kamogawa city, Chiba1 (35°07'48.4"N 139°58'27.7"E) (16 May 2014)	22.6	9.0	sen0301, sen0402	*S.* sp. JPS002		
			senA1001, senA2004, sen01401, senB2002	*S.* sp. JPS015		
			senA1602	*S.* sp. JPS038	MK558108	MK558180
			senA2302, senA2305, senB2003, senB2005, senB2605	*S.* sp. JPS038		
			senA2002	*S.* sp. JPS039	MK558111	MK558150
			senA2303	*S.* sp. JPS040	MK558138	MK558172
			senB2001	*S.* sp. JPS041	MK558142	MK558185
			senB2004	*S.* sp. JPS041		
			senB2603	*S.* sp. JPS042	MK558120	MK558155
			senB2604	*S.* sp. JPS043	MK558122	MK558145
			sen0102	*S.* sp. JPS044	MK558110	MK558149
			sen0502	*S.* sp. JPS044		
			sen0103	*S.* sp. JPS045	MK558129	MK558181
			sen01505	*S.* sp. JPS046	MK558130	MK558191
			sen0406	*S.* sp. JPS047	MK558125	MK558156
A narrow stream	14.2	7.6	kit0101	*S.* sp. JPS027	MK558118	MK558154

in Kitayama park,<br>Toyko<br>(35°45'59.9"N<br>139°27'31.2"E)<br>(6 April 2015)						
			kit0201 [=NIES-4307]	*S. longata* (Vaucher) Kützing JPS006	MK558124	MK558143
			kit0301, kit0401	*S.* sp. JPS006		
			kit2	*S.* sp. JPS028	MK558094	MK558162
			kit42B	*S.* sp. JPS029	MK558096	MK558159
Paddy fields in<br>Yasu city, Shiga<br>(35°05'31.9"N<br>136°04'21.9"E)<br>(8 April 2015)	9.0	8.1	shi1101	*S.* sp. JPS021		
			shi1701	*S.* sp. JPS038		
			shi0303	*S.* sp. JPS048	MK558131	MK558182
			shi0305 [=NIES-4311]	*S. mirabilis* (Hassall) Kützing JPS010	MK558133	MK558174
			shi0308	*S.* sp. JPS049	MK558093	MK558166
Lake Biwa, Shiga1<br>(35°04'17.7"N<br>135°56'18.3"E)<br>(1 May 2015)	29.9	6	biw0302 [=NIES-4305]	*S. hopeiensis* Jao JPS004	MK558112	MK558147
			biw0601	*S.* sp. JPS017	MK558114	MK558146
			biw0602	*S.* sp. JPS018	MK558107	MK558177
			biw0604	*S.* sp. JPS019	MK558117	MK558148
			biw0703	*S.* sp. JPS021	MK558119	MK558153
			biw0101, biw0201, biw0501, biw0802m, biw0802f	*S.* sp. JPS008		
A pond in<br>Takamatsu city,<br>Kagawa<br>(34°19'21.8"N<br>134°02'05.8"E)<br>(27 May 2015)	25.3	9.4	taka0101	*S.* sp. JPS051	MK558095	MK558163
			taka1201	*S.* sp. JPS052	MK558139	MK558168
Lake Biwa, Shiga2<br>(35°03'10.5"N<br>135°52'32.0"E)<br>(30 June 2015)	29.8	9.1	biw06300601	*S.* sp. JPS020	MK558128	MK558173
			biw160601	*S.* sp. JPS022	MK558097	MK558194
			biw160602	*S.* sp. JPS023	MK558101	MK558160
			biw160604	*S.* sp. JPS028		
Paddy fields in<br>Kamogawa city,<br>Chiba2<br>(35°07'51.7"N<br>139°58'31.2"E)<br>(15 April 2016)	25.4	7.8	chi0101	*S.* sp. JPS024	MK558091	MK558165
			chi0102 [=NIES-4316]	*S. varians* (Hassall) Kützing JPS015	MK558105	MK558178
			chi0202 [=NIES-4308]	*S. majuscula* Kützing JPS007	MK558140	MK558184
			chi0305 [=NIES-4313]	*S. pseudomaxima* Kadłubowska JPS012	MK558102	MK558161
			chi0504	*S.* sp. JPS025	MK558132	MK558187
			chi0207	*S.* sp. JPS009		
			chi0802	*S.* sp. JPS021		
Paddy fields in<br>Kamogawa city,<br>Chiba3<br>(35°08'03.2"N<br>139°59'00.5"E)<br>(28 Febrary 2017)	13.8	6.2	chiA101[=NIES-4304]	*S. dentireticulata* Jao JPS003	MK558115	MK558152
			chiA102	*S.* sp. JPS015		
			chiA301	*S.* sp. JPS028		
			chiA302	*S.* sp. JPS024		
			chiA304 [=NIES-4315]	*S. semiornata* Jao JPS014	MK558116	MK558144
			chiA305 [=NIES-4306]	*S. longata* (Vaucher) Kützing JPS005	MK558123	MK558157
			chiA307 [=NIES-4312]	*S. pratensis* Transeau JPS011	MK558134	MK558193

			chiA308	*S.* sp. JPS011		
			chiA401	*S.* sp. JPS026	MK558106	MK558192
A pond in Obuse Town, Nagano (36°41'27"N 138°19'49"E) (28 March 2017)	11.6	7	nag101 [=NIES-4310]	*S. minuticrassoides* Yamagishi JPS009	MK558098	MK558164
A pond in Nagano city, Nagano1 (36°37'04.40"N 138°16'25"E) (30 March 2017)	11.8	7	nag201	*S.* sp. JPS032	MK558109	MK558179
A pond in Nagano city, Nagano2 (36°37'04.40"N 138°16'25"E) (30 March 2017)	10.0	7	nag301 [=NIES-4309]	*S. majuscula* Kützing JPS008	MK558141	MK558183
A pond in Suzaka city, Nagano (36°37'47.4"N 138°17'22.7"E) (30 March 2017)	13.4	7	nag401	*S.* sp. JPS033		
Pond Ochikage-ike, Nagano (36°46'56.9"N 138°12'59.0"E) (18 May 2017)	-	-	nag501	*S.* sp. JPS008		
			nag503	*S.* sp. JPS028		
			nag504	*S.* sp. JPS033	MK558092	MK558190
Pond Tamizo-ike, Nagano (36°16'28.66"N 137°58'05.72"E) (6 June 2017)	24.9	-	nag701	*S.* sp. JPS034	MK558100	MK558188
			nag702	*S.* sp. JPS035	MK558099	MK558189
			nag703	*S.* sp. JPS028		
			nag704	*S.* sp. JPS007		
			nag706	*S.* sp. JPS036	MK558121	MK558158
			nag707	*S.* sp. JPS037	MK558137	MK558171
Paddy fields in Matsumoto city, Nagano (36°12'40.91"N 137°59'25.53"E) (6 June 2017)	30.0	-	nag901	*S.* sp. JPS028		
Lake Nojiri, Nagano1 (36°49'59.97"N 138°14'32.71"E) (28 July 2017)	25.9	-	nagXII04	*S.* sp. JPS005		
			nagX03, nagXI02, nagXII03, nagXIII03	*S.* sp. JPS013		
Lake Nojiri, Nagano2 (36°49'59.97"N 138°14'32.71"E) (23 August 2017)	26.1	-	nagXV01	*S.* sp. JPS034		
			nagXIV03, nagXV04	*S.* sp. JPS013		
Pond Obasute-ooike, Nagano (36°29'17.03"N 138°04'59.82"E) (3 September 2017)	21.5	-	nagXVI01	*S.* sp. JPS013		
Lake Hijiri, Nagano (36°29'18.32"N 138°03'55.14"E) (3 September 2017)	23.1	-	nagXVIII01	*S.* sp. JPS040		
			nagXVIII02	*S.* sp. JPS001		
Pond Gongen-ike, Nagano	18.2	-	nagXIX04	*S.* sp. JPS005		

(36°29'36.24"N 138°01'20.05"E) (3 September 2017)			nagXIX06	*S.* sp. JPS006
Pond Daizahoushi-ike, Nagano (36°42'22.54"N 138°08'86.91"E) (11 September 2017)	22.8	-	nagXXI02	*S.* sp. JPS007
			nagXX01, nagXX03, nagXXI01,	*S.* sp. JPS034
			nagXX02	*S.* sp. JPS028
Lake Reisenji, Nagano (36°44'51.53"N 138°11'04.69"E) (11 September 2017)	22.7	-	nagXXIV01, nagXXIV04	*S.* sp. JPS031

*Fifty-two strains with different *rbcL*-types (underlined) were selected for conjugation experiments. Note that the 122 strains exhibit 52 different *rbcL* sequences or types.

**Scientific name with specific epithet represents a taxonomic species that was identified based on vegetative and reproductive characteristics observed under the present cultural conditions (Figs. 1-4). Taxonomically unidentified species (*S.* sp.) were distinguished and labeled based on their *rbcL*-types.

***Species that may belong to *Temnogyra* or *Sirogonium* because of lack of sexual reproduction characteristics.

Supplementary Table S2. List of primers used for sequencing *rbcL* genes.

Designation	Positions*	sequence (5'-3')
CHAR-RF-1**	1-23	ATGTCACCACAGACAGAAACTAA
CHAR-RF-2**	560-581	GAGCTCTATATGAATGTCTTCG
CHAR-RR-3**	695-676****	GTTTCTGCTTGAGATTTATA
R42AE***	1403-1384****	TC(AG)AA(CT)TT(AG)AT(CT)TC(CT)TTCCA

*Coordinate number from the *Chara connivens* Salzmann ex A. Braun *rbcL* gene[S1].

**Primers from Sakayama *et al.*[S2].

***Degenerate primer from Shimada *et al*.[S3].

****Reverse primer.

Supplementary Table S3. List of primers used for sequencing *atpB* genes.

Designation	Positions*	sequence (5'-3')
atpB-175FZYG**	174-194	TRTWACYTGTGARGTACARCA
atpB-700F**	700-719	TATGGTCAAATGAATGAACC
atpB-866R**	884-866***	CCWACTGCAGAAGGCATAC
atpB-1404RZYG**	1423-1402***	CYARRTARAACGCYTGTTCTGG

*Coordinate number from the *Spirogyra maxima* (Hassall) Wittrock gene[S4].

**Primers from Stancheva *et al.*[S5].

***Reverse primer.

Supplementary Table S4. List of additional *rbcL* and *atpB* gene sequences to Table S1, included in the present phylogenetic analysis.

Strain	ingroup/ outgroup	Species	*rbcL*	*atpB*
ACOI 901	ingroup	*Actinotaenium cucurbitinum* (Biss.) Teiling	EF371279	KC779063
JH0199	ingroup	*Bambusina borreri* (Ralfs) Cleve	EF371283	KC779064
UTEX 1075	ingroup	*Closterium acerosum* Ehr. ex Ralfs	EF371285	KC779065
JH0013	ingroup	*Closterium ehrenbergii* var. *malinvernianum* (De Notaris) Rabenh.	EF371286	KC779066
JH0021	ingroup	*Closterium libellula* Focke	EF371287	KC779067
CFD300a1	outgroup	*Coleochaete divergens* Pringsh.	AY082330	AY082326
UTEX 1261	outgroup	*Coleochaete nitellarum* Jost	AY051140	AY082325
CFD57b6	outgroup	*Coleochaete pulvinata* A. Braun ex. Kütz.	AY082310	AY082307
SAG 3.90	outgroup	*Coleochaete scutata* Bréb.	AY082329	AY082324
CFD10d1	outgroup	*Coleochaete sieminskiana* Szymanska	AF408249	AF408791
UTEX 301	ingroup	*Cosmarium botrytis* Menegh.ex Ralfs	EF371288	KC779068
ACOI 95	ingroup	*Cosmocladium saxonicum* De Bary	EF371292	KC779069
UTEX 1259	ingroup	*Cylindrocystis brebissonii* (Ralfs) De Bary	EF371293	KC779070
JH0038	ingroup	*Cylindrocystis* sp.	EF371296	KC779071
UTEX 1925	ingroup	*Cylindrocystis* sp.	EF371295	KC779072
SVCK 108	ingroup	*Desmidium aptogonum* Bréb. ex Archer	EF463091	KC779073
SVCK 113	ingroup	*Desmidium grevillei* (Kütz.) De Bary	EF463090	KC779074
JH0018	ingroup	*Euastrum crassum* var. *michiganense* Prescott	EF371300	KC779075
ACOI 350	ingroup	*Gonatozygon kinahani* (Archer) Rabenh.	AJ553945	KC779076
SVCK 158	ingroup	*Gonatozygon monotaenium* De Bary	KC779163	KC779077
SVCK 302	ingroup	*Haplotaenium minutum* (Ralfs) Bando	EF371326	KC779078
SVCK 428	ingroup	*Heimansia pusilla* (Hilse) Coesel	EF371291	KC779079
SAG 384.2	ingroup	*Hyalotheca dissiliens* (Smith) Bréb. ex Ralfs	KC779164	KC779080
UTEX 41	ingroup	*Mesotaenium caldariorum* (Lagerheim) Hansg.	EF371307	AF408798
UTEX 1024	ingroup	*Mesotaenium kramstai* Lemmerm.	EF371309	KC779082
JH0031	ingroup	*Mesotaenium* sp.	EF371310	KC779081
UTEX 1941	ingroup	*Micrasterias rotata* Ralfs	EF371312	KC779083
UTEX 758	ingroup	*Mougeotia* sp.	AF408252	AF408800
UTEX 561	ingroup	*Netrium digitus* (Bréb. ex Ralfs) Itzigsohn & Rothe	U38698	KC779084
ACOI 780	ingroup	*Penium cylindrus* Bréb. ex Ralfs	EF371320	KC779085
ACOI 330	ingroup	*Penium maragaritaceum* Bréb. ex Ralfs	EF371322	KC779086
SAG 47.89	ingroup	*Phymatodocis nordstedtianum* Wolle	KC779165	KC779087
ARL 700	ingroup	*Sirogonium melanosporum* (Randhawa) Transeau	L13484	KC779088
UTEX 1985	ingroup	*Sirogonium sticticum* (Sm.) Kütz.	DQ015924	KC779089
RSS007	ingroup	*Spirogyra borgeana* Transeau	KC779194	KC779128
RSS024	ingroup	*Spirogyra borgeana* Transeau	KC779209	KC779138
RSS025	ingroup	*Spirogyra borgeana* Transeau	KC779210	KC779139
RSS001	ingroup	*Spirogyra californica* Stancheva, J. D. Hall, McCourt et Sheath *sp. nov.*	KC779188	KC779123
UTEX 2462	ingroup	*Spirogyra communis* (Hassall) Kütz.	DQ015932	KC779090
UTEX 1744	ingroup	*Spirogyra condensata* (Vaucher) Kütz.	DQ015936	KC779091
JH0425	ingroup	*Spirogyra croasdaleae* Blum	KC779170	KC779107
RSS033	ingroup	*Spirogyra fluviatilis* Hilse	KC779218	KC779146
UTEX 1743	ingroup	*Spirogyra gracilis* (Hassall) Kütz.	DQ015937	KC779092
RSS011	ingroup	*Spirogyra grevilleana* (Hassall) Kütz.	KC779197	KC779130
RSS017	ingroup	*Spirogyra grevilleana* (Hassall) Kütz.	KC779202	KC779133
UTEX 477	ingroup	*Spirogyra grevilleana* (Hassall) Kütz.	DQ015938	KC779093
UTEX 1742	ingroup	*Spirogyra juergensii* Kütz.	DQ015939	KC779094

RSS015	ingroup	*Spirogyra juliana* Stancheva, J. D. Hall, McCourt et Sheath *sp. nov.*	KC779200	KC779131
UTEX 1745	ingroup	*Spirogyra liana* Transeau	DQ015940	KC779095
RSS031	ingroup	*Spirogyra longata* (Vaucher) Kütz.	KC779216	KC779144
RSS003	ingroup	*Spirogyra lutetiana* P. Petit	KC779190	KC779125
RSS018	ingroup	*Spirogyra lutetiana* P. Petit	KC779203	KC779134
RSS006	ingroup	*Spirogyra majuscula* Kütz.	KC779193	KC779127
RSS026	ingroup	*Spirogyra maxima* (Hassall) Kütz.	KC779211	KC779140
RSS032	ingroup	*Spirogyra maxima* (Hassall) Kütz.	KC779217	KC779145
UTEX 2495	ingroup	*Spirogyra maxima* (Hassall) Kütz.	DQ015941	AF408797
RSS008	ingroup	*Spirogyra notabilis* Taft	KC779195	KC779129
JH0002	ingroup	*Spirogyra parvula* (Transeau) Czurda	KC779166	KC779101
UTEX 1746	ingroup	*Spirogyra pratensis* Transeau	DQ015949	KC779098
UTEX 928	ingroup	*Spirogyra pratensis* Transeau	DQ015948	KC779097
JH0058	ingroup	*Spirogyra* sp.	DQ015954	KC779102
JH0130	ingroup	*Spirogyra* sp.	DQ015955	KC779104
JH0263	ingroup	*Spirogyra* sp.	KC779168	KC779105
JH0278	ingroup	*Spirogyra* sp.	KC779169	KC779106
JH0429	ingroup	*Spirogyra* sp.	KC779171	KC779108
JH0643	ingroup	*Spirogyra* sp.	KC779173	KC779110
JH0728	ingroup	*Spirogyra* sp.	KC779174	KC779111
JH0744	ingroup	*Spirogyra* sp.	KC779175	KC779112
JH0763	ingroup	*Spirogyra* sp.	KC779176	KC779113
JH0941	ingroup	*Spirogyra* sp.	KC779179	KC779115
JH0977	ingroup	*Spirogyra* sp.	KC779182	KC779118
JH0979	ingroup	*Spirogyra* sp.	KC779183	KC779119
JH0987	ingroup	*Spirogyra* sp.	KC779185	KC779121
RSS020	ingroup	*Spirogyra* sp.	KC779205	KC779135
RSS036	ingroup	*Spirogyra* sp.	KC779220	KC779148
RSS021	ingroup	*Spirogyra submaxima* Transeau	KC779206	KC779136
ACOI 1925	ingroup	*Spirogyra tenuissima (Hassall)* Kütz.	KC779187	KC779099
JH1015	ingroup	*Spirogyra tenuissima (Hassall)* Kütz.	KC779186	KC779122
RSS027	ingroup	*Spirogyra teodoresci* Transeau	KC779212	KC779141
UTEX 479	ingroup	*Spirogyra varians* (Hassall) Kütz.	DQ015951	KC779100
RSS004	ingroup	*Spirogyra weberi* Kütz.	KC779191	KC779126
SVCK 365	ingroup	*Spondylosium pulchellum* (Archer) Archer	AM911261	KC779153
JH0014	ingroup	*Staurastrum arctiscon* (Ehr.) Lundell	EF371343	KC779154
SAG 679.1	ingroup	*Staurastrum punctulatum* Bréb.	FN432117	NC_008116
UTEX 2508	ingroup	*Staurodesmus convergens* (Ehrenb.) Lillieroth	EF371281	KC779155
SAG 25.88	ingroup	*Teilingia granulata* (Roy & Bissett) Bourr.	EF371350	KC779156
JH0054	ingroup	*Xanthidium antilopaeum* var. *polymazum* Nordst.	EF378638	KC779157
SAG 698-2	ingroup	*Zygnema cylindricum* Transeau	EF371357	KC779158
JH0007	ingroup	*Zygnema* sp.	EF371359	KC779159
JH0044	ingroup	*Zygnema* sp.	JF965517	KC779160
ACOI 60	ingroup	*Zygnemopsis minuta* Randhawa	EF371363	KC779161
UTCC 136	ingroup	*Zygogonium tunetanum* Gauth.-Lièvre	JQ780057	KC779162

Supplementary Table S5. Information of vegetative cells about 52 strains in this study.

Strain (*rbcL*-type)	Species	Width (μm)	Length (μm)	End wall	Chloroplasts per cell	Turns per cell
Uki1 [=NIES-4302] (JPS001)	*S. chungkingensis* Jao	24-27	150-300	plane	3	4-5
A2F [=NIES-4303] (JPS002)	*S. corrugata* Transeau	28-34	200-600	plane	2-3	3-5
chiA101 [=NIES-4304] (JPS003)	*S. dentireticulata* Jao	20-24	200-340	replicate	1	4-5
biw0302 [=NIES-4305] (JPS004)	*S. hopeiensis* Jao	24-28	110-260	replicate	1	5-6
chiA305 [=NIES-4306] (JPS005)	*S. longata* (Vaucher) Kützing	26-30	110-260	plane	1	4-5
kit0201 [=NIES-4307] (JPS006)	*S. longata* (Vaucher) Kützing	27-32	110-250	plane	1	3-6
chi0202 [=NIES-4308] (JPS007)	*S. majuscula* Kützing	70-80	170-300	plane	6	1.5-2
nag301 [=NIES-4309] (JPS008)	*S. majuscula* Kützing	70-80	110-270	plane	6	1-2
nag101 [=NIES-4310] (JPS009)	*S. minuticrassoides* Yamagishi	95-112	295-515	plane	6	2.5-3
shi0305 [=NIES-4311] (JPS010)	*S. mirabilis* (Hassall) Kützing	20-26	105-270	plane	1	6-8
chiA307 [=NIES-4312] (JPS011)	*S. chenii* Jao	17-22	160-270	plane	1	3-5
chi0305 [=NIES-4313] (JPS012)	*S. pseudomaxima* Kadłubowska	135-140	190-430	plane	7-8	1-1.5
Tpx8 [=NIES-4314] (JPS013)	*S. punctata* Cleve	26-30	150-300	plane	1	5-7
chiA304 [=NIES-4315] (JPS014)	*S. semiornata* Jao	26-32	250-380	replicate	1	5-6
chi0102 [=NIES-4316] (JPS015)	*S. varians* (Hassall) Kützing	28-33	125-200	plane	1	4-8
A3 (JPS016)	*S.* sp.*	10-12	90-180	plane	1	4-5
biw0601 (JPS017)	*S.* sp.	15-20	210-350	replicate	1	5-7
biw0602 (JPS018)	*S.* sp.	24-30	120-190	plane	1	3-5
biw0604 (JPS019)	*S.* sp.	8-12	120-160	replicate	1	4-7
biw06300601 (JPS020)	*S.* sp.	21-25	170-300	plane	1	5-7
biw0703 (JPS021)	*S.* sp.	42-52	32-590	plane	3-4	2-4
biw160601 (JPS022)	*S.* sp.	-	-	-	-	-
biw160602 (JPS023)	*S.* sp.	135-148	400-610	plane	5	2-3
chi0101 (JPS024)	*S.* sp.	22-28	110-270	plane	2	3-5
chi0504 (JPS025)	*S.* sp.	20-25	80-130	plane	1	3-4
chiA401 (JPS026)	*S.* sp.	22-30	105-240	plane	1	4-5
kit0101 (JPS027)	*S.* sp.	13-16	125-185	replicate	1	4-5
kit2 (JPS028)	*S.* sp.	66-88	140-240	plane	3	1.5-2
kit42B (JPS029)	*S.* sp.	32-45	110-180	plane	3	2-3
mit0203 (JPS030)	*S.* sp.	10-17	30-115	plane	1	3-4
mitA01 (JPS031)	*S.* sp.	-	-	-	-	-
nag201 (JPS032)	*S.* sp.	22-28	120-240	plane	1	3-5

nag504 (JPS033)	*S.* sp.	-	-	-	-	-
nag701 (JPS034)	*S.* sp.	132-142	285-440	plane	6-7	3-4
nag702 (JPS035)	*S.* sp.	-	-	-	-	-
nag706 (JPS036)	*S.* sp.	30-42	115-340	plane	1	3-6
nag707 (JPS037)	*S.* sp.	51-57	130-280	plane	3-4	2-3
senA1602 (JPS038)	*S.* sp.	20-23	75-150	plane	1	1-3
senA2002 (JPS039)	*S.* sp.	14-19	185-300	replicate	1	5-7
senA2303 (JPS040)	*S.* sp.	45-55	215-320	plane	4	2-5
senB2001 (JPS041)	*S.* sp.	64-74	190-360	plane	4	1-3
senB2603 (JPS042)	*S.* sp.	19-25	250-400	replicate	1	5-7
senB2604 (JPS043)	*S.* sp.	18-24	250-540	replicate	1	4-6
sen0102 (JPS044)	*S.* sp.	12-15	200-380	replicate	1	4-7
sen0103 (JPS045)	*S.* sp.	14-18	94-340	plane	1	4-7
sen01505 (JPS046)	*S.* sp.	20-26	150-210	plane	1	5-6
sen0406 (JPS047)	*S.* sp.	22-28	280-410	plane	1	4-8
shi0303 (JPS048)	*S.* sp.	13-20	110-230	plane	1	4-6
shi0308 (JPS049)	*S.* sp.	39-44	160-250	plane	3	2-4
T11 (JPS050)	*S.* sp.	11-14	110-195	replicate	1	3-5
tak0101 (JPS051)	*S.* sp.	80-86	100-140	plane	4-5	1-2
tak1201 (JPS052)	*S.* sp.	36-47	200-510	plane	1	6-7

* Species that may belong to *Temnogyra* or *Sirogonium* because of lack of sexual reproduction characteristics.

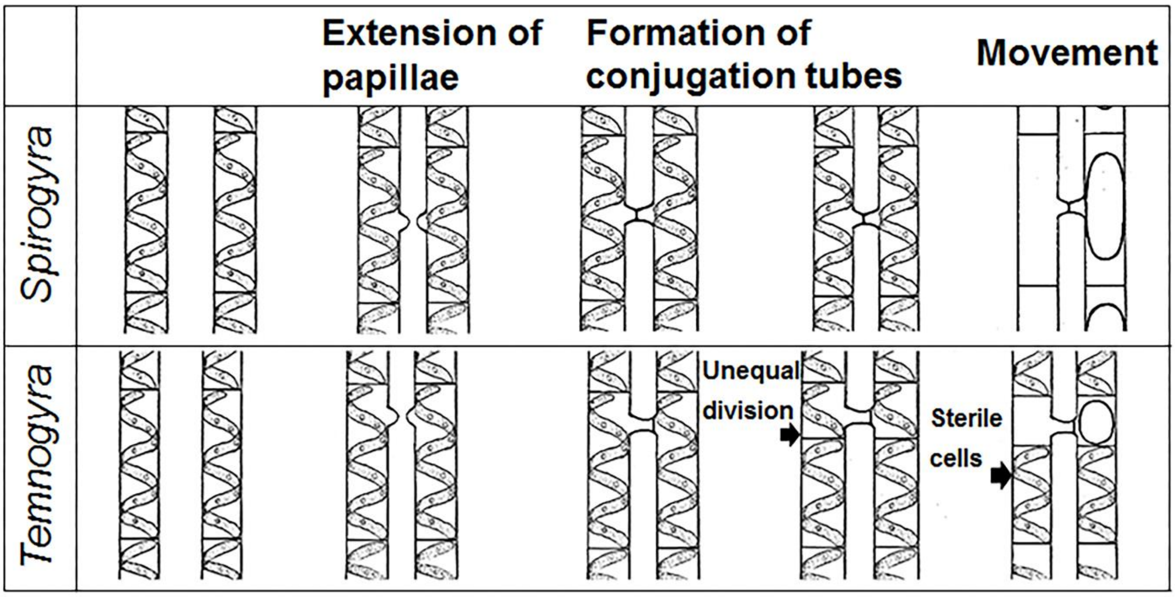

Supplementary Figure S1. Comparison of conjugation processes in *Spirogyra* and *Temnogyra*. Based on Yamagishi[S6].

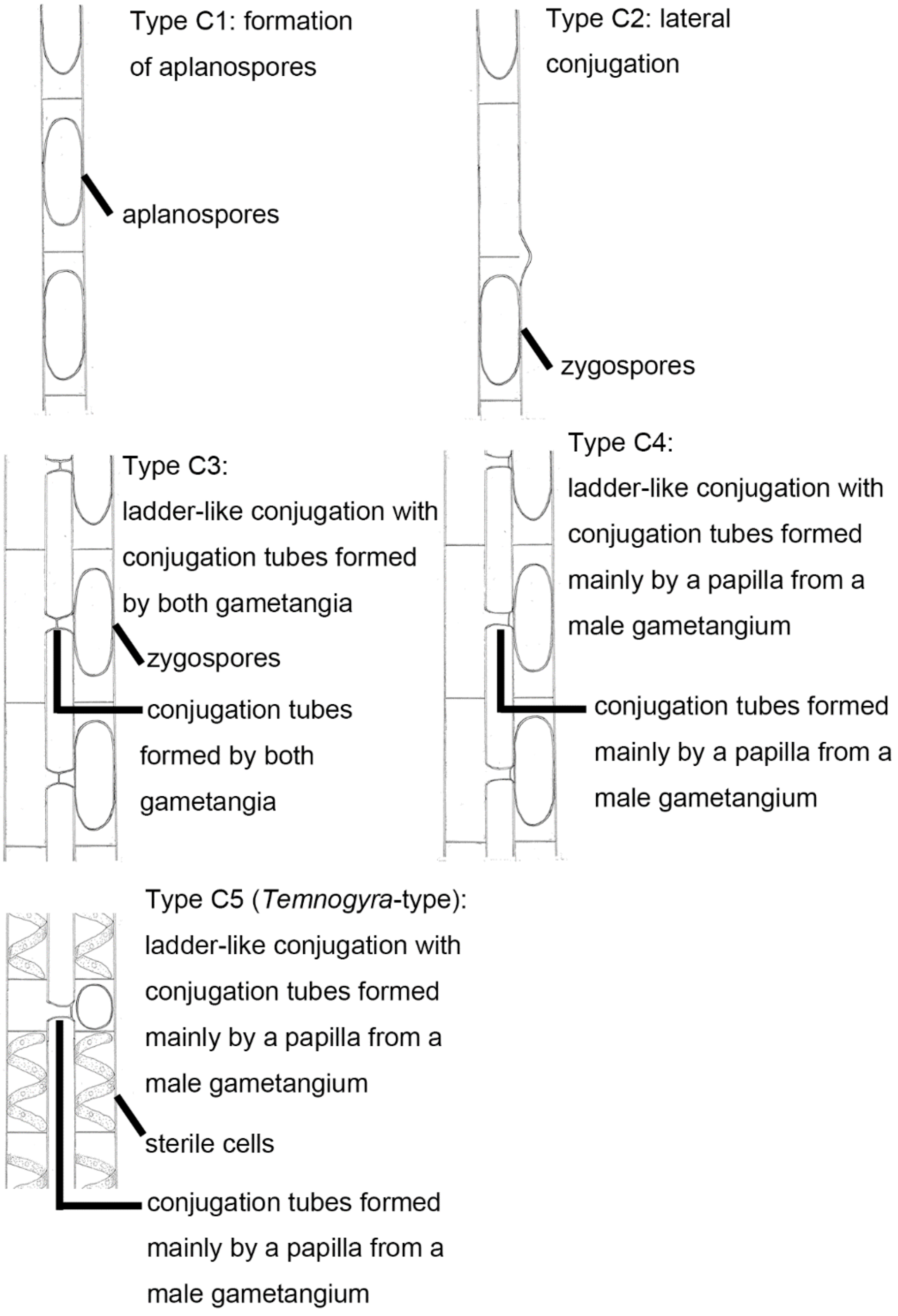

Supplementary Figure S2. Diagrams of five types of formation of zygospores or aplanospore observed in this study.

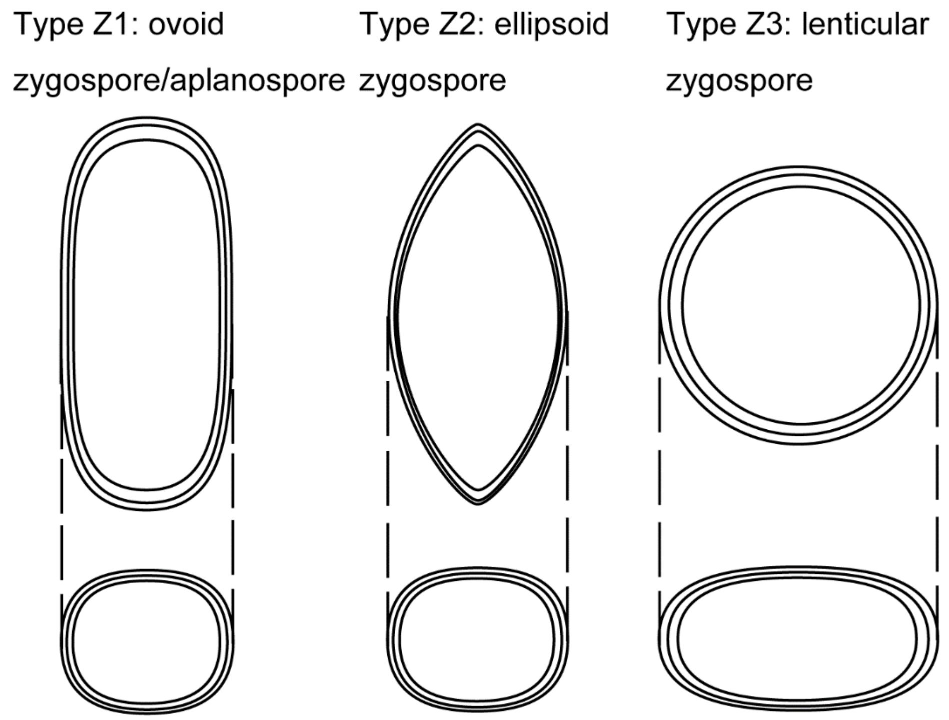

Supplementary Figure S3. Diagrams of three types of zygospores or aplanospores observed in this study. Terminology is based on Transeau[S7].

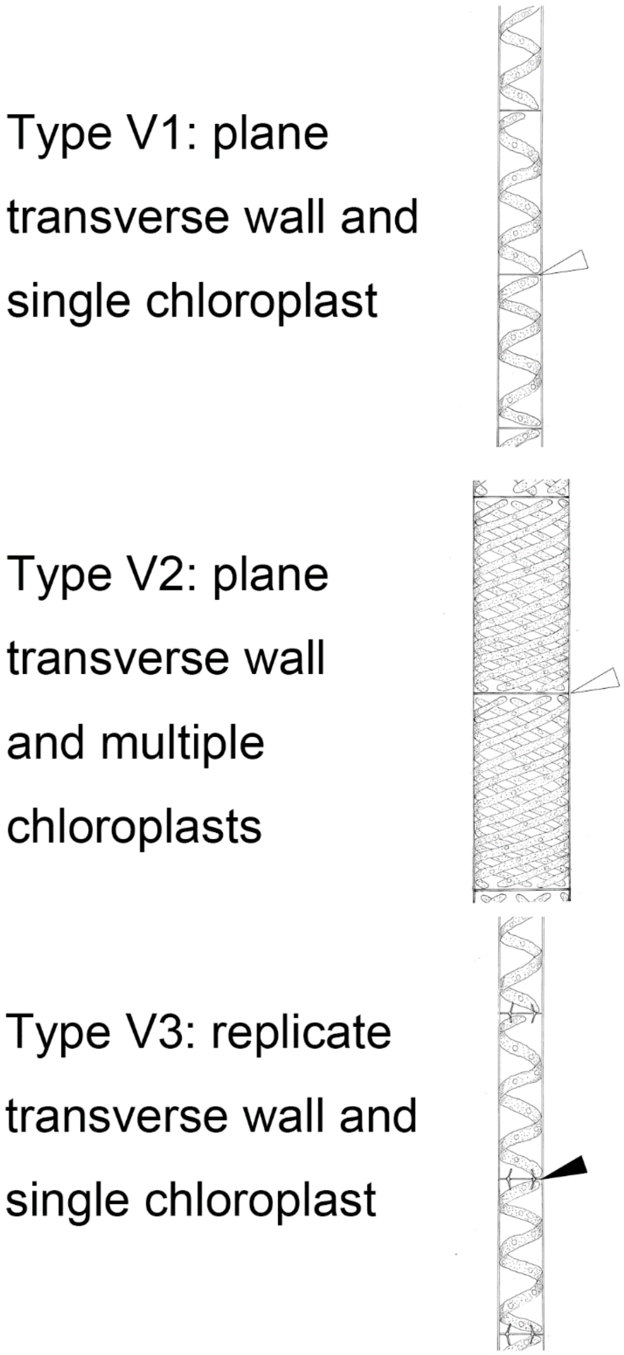

Supplementary Figure S4. Diagrams of three types of vegetative cells observed in this study. The open arrowheads show plane transverse wall and the closed arrowhead shows replicate transverse wall.

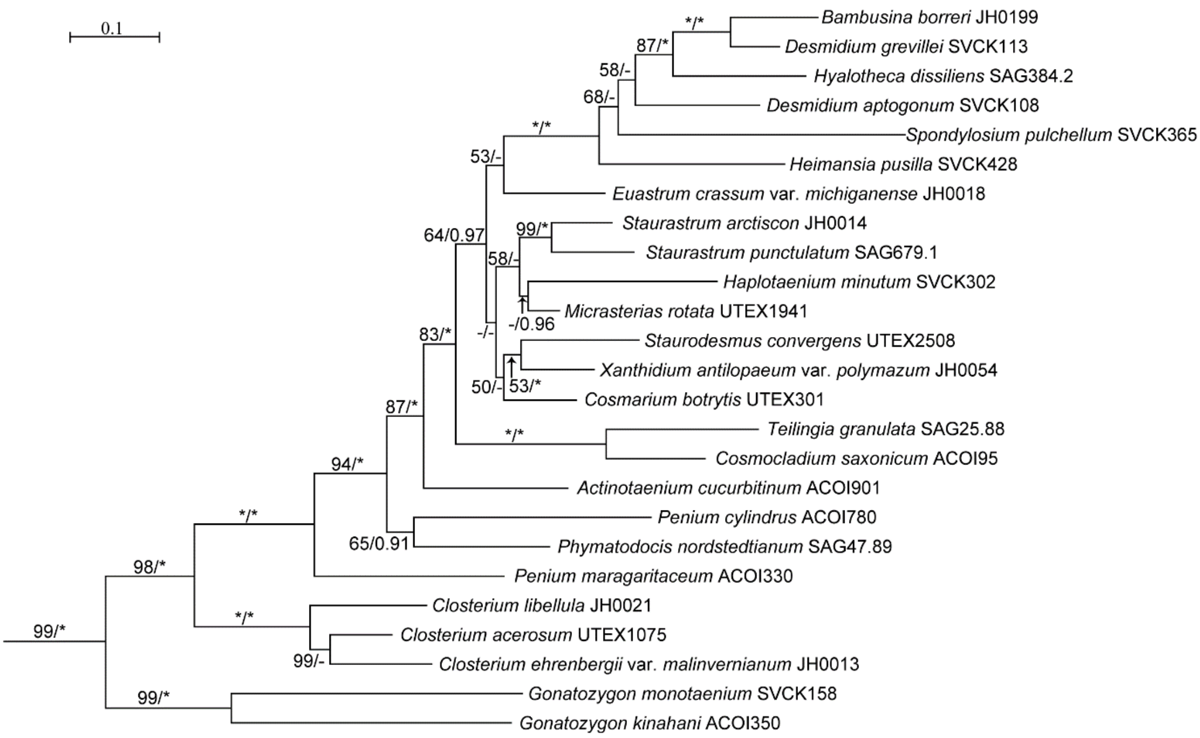

Supplementary Figure S5. Details of “Desmidiales” in ML tree (Fig. 5a). For details of the explanation of the tree, see Figure 5.

Supplementary Notes

Supplementary Note S1. Taxonomic accounts

Spirogyra chungkingensis Jao 1935: 600[S8]. (Figs. 2a-d)

Species description: Vegetative cells 24-27 μm wide, 150-300 μm long; transverse walls plane; chloroplasts 3 per cell (Fig. 2a). Conjugation scalariform (like ladder); conjugation tubes formed by both gametangia; fertile female gametangia slightly inflated (Fig. 2b). Zygospores ellipsoid, 33-35 × 55-83 μm; mesospore two-layered, outer brownish, wrinkled; inner brown, irregularly reticulate (Figs. 2c, d).

Distribution: China and Japan[S6,S9] (Table S1).

Strain examined: Uki1 (JPS001).

Remarks: *Spirogyra chungkingensis* was originally described by Jao[S8] based on the field-collected material from China. The present Japanese material agrees with the species concept of *S. chungkingensis* by Jao[S8] in having plane transverse walls, 3 chloroplasts and two-layered mesospores, of which the outer is thin and wrinkled, the inner irregularly reticulate (Figs. 2c, d).

Spirogyra corrugata Transeau 1934: 229[S10]. (Figs. 4a-e)

Synonym: Temnogyra corrugata (Transeau) Yamagishi 1963: 207[S11].

Species description: Vegetative cells 28-34 μm wide, 200-600 μm long; transverse walls plane; chloroplasts 2-3 per cell (Fig. 4a). Conjugation scalariform; conjugation tubes formed by male gametangia; fertile female gametangia inflated (Fig. 4b). Gametangia adjoining sterile cells in both male and female filaments (Fig. 4b). Zygospores ovoid, 50-54 × 97-111 μm; mesospore two-layered, the outer thin, corrugate and the inner thick, reticulate (Figs. 4d, e).

Distribution: United States, China, Africa, and Japan[S6,S7,S9,S12-14] (Table S1).

Strain examined: A2F (JPS002).

Remarks: This species was originally described by Transeau[S10] as *Spirogyra corrugata*, and was transferred to *Temnogyra*, as *Temnogyra corrugata* by Yamagishi[S11] based on the presence of sterile cells adjoining gametangia. However, no subsequent authors recognize the genus *Temnogyra*. Transeau[S7] described morphological details of this species such as having plane transverse walls, 1-3 chloroplasts, and two-layered mesospores. The present Japanese material agrees with the species concept of *T. corrugate* by Transeau[S7]. The present phylogenetic analysis demonstrated that this species is not separated from species of

Spirogyra and *Sirogonium* sensu Yamagishi[S11]. Thus, this species should be classified to the genus *Spirogyra*.

Spirogyra dentireticulata Jao 1935: 611[S8]. (Figs. 1a-d)

Species description: Vegetative cells 20-24 μm wide, 200-340 μm long; transverse walls replicate; chloroplasts single per cell (Fig. 1a). Conjugation scalariform; conjugation tubes formed by both gametangia; fertile female gametangia slightly enlarged (Fig. 1b). Zygospores ovoid, 30-34 × 70-76 μm; mesospore yellow-brown, reticulate with coarse ridges (Figs. 1c, d).

Distribution: China, USA, Kazakhstan and Japan[S7,S9,S13] (Table S1).

Strain examined: chiA101 (JPS003).

Remarks: *Spirogyra dentireticulata* was originally described by Jao[S8] based on the field-collected material from China. The present Japanese material agrees with the species concept of *S. dentireticulata* by Jao[S8] and Transeau[S7] in having replicate transverse walls, single chloroplasts and reticulate mesospore with spinulose-dentate reticulations at the intersections. Although Jao[S8] and Transeau[S7] reported both lateral and scalariform conjugation in this species, we observed only scalariform conjugation of Japanese material under the present culture conditions. This species has not previously been recorded from Japan[S6].

Spirogyra hopeiensis Jao 1935: 608[S8]. (Figs. 1e-h)

Species description: Vegetative cells 24-28 μm wide, 110-260 μm long; transverse walls replicate; chloroplasts single per cell (Fig. 1e). Conjugation scalariform; conjugation tubes formed by male gametangia; fertile female gametangia inflated mostly on the conjugation sides (Fig. 1f). Zygospores ellipsoid, 30-34 × 59-62 μm; mesospore yellow-brown, smooth (Figs. 1g, h).

Distribution: Kazakhstan, China and Japan[S6,S9,S13] (Table S1).

Strain examined: biw0302 (JPS004).

Remarks: *Spirogyra hopeiensis* was originally described by Jao[S8] based on the field-collected material from China. The present cultured material agrees with the description of *S. hopeiensis* by Jao[S8] and Yamagishi[S6] in having replicate transverse walls, single chloroplasts, conjugation tubes formed by male gametangia, female gametangia inflated on the inner side and smooth single-layered mesospore.

Spirogyra longata (Vaucher) Kützing 1843: 279[S15]. (Figs. 1i-l)

Basionym: Conjugata longata Vaucher 1803: 71[S16].

Species description: Vegetative cells 26-32 μm wide, 110-260 μm long; transverse walls plane; chloroplasts single per cell (Fig. 1i). Conjugation scalariform; conjugation tubes formed by both gametangia; fertile female gametangia cylindrical, sometimes slightly enlarged (Fig. 1j). Zygospores ellipsoid to ovoid, 28-32 × 48-74 μm; mesospore yellow-brown, smooth (Figs. 1k, l).

Distribution: Widely distributed in the United States, Europe, China and Japan[S6,S7,S9,S12-14] (Table S1).

Strain examined: chiA305 (JPS005) and kit0201 (JPS006).

Remarks: *Spirogyra longata* was originally described by Vaucher[S16] as *Conjugata longata*, and was transferred to *Spirogyra*, as *Spirogyra longata* by Kützing[S15]. Transeau[S7] described morphological details of this species such as having plane transverse walls, single chloroplasts, cylindrical female gametangia and smooth mesospores. The present Japanese material agrees with the species concept of *S. longata* by Transeau[S7]. Although Transeau[S7] reported both lateral and scalariform conjugation in this species, we observed only scalariform conjugation of Japanese material under the present culture conditions.

Spirogyra majuscula Kützing 1849: 441[S17]. (Figs. 3a-d)

Species description: Vegetative cells 70-80 μm wide, 110-300 μm long; transverse walls plane; chloroplasts 6 per cell (Fig. 3a). Conjugation scalariform; conjugation tubes formed by both gametangia; fertile female gametangia slightly inflated on the outer side (Fig. 3b). Zygospores lenticular, 58-78 × 58-78 × 43-55 μm; mesospore brown, smooth (Figs. 3c, d).

Distribution: Widely distributed in Europe, America, Asia, Africa, and Australia[S6,S7,S9,S12-14] (Table S1).

Strain examined: chi0202 (JPS007) and nag301 (JPS008).

Remarks: *Spirogyra majuscula* was originally described by Kützing[S17]. Transeau[S7] characterized this species by detailed vegetative and reproductive morphology based on the field-collected material from United States. The present Japanese material agrees with the species concept of *S. majuscula* by Transeau[S7] in having plane transverse walls, 5-8 chloroplasts and smooth mesospore. Although Transeau[S7] reported both lateral and scalariform conjugation in this species, we observed only scalariform conjugation of Japanese material under the present culture conditions.

Spirogyra minuticrassoidea Yamagishi 1963: 216[S18]. (Figs. 3e-h)

Species description: Vegetative cells 95-112 μm wide, 295-515 μm long; transverse walls plane; chloroplasts 6 per cell (Fig. 3e). Conjugation scalariform; conjugation tubes formed by both gametangia; fertile female gametangia cylindrical (Fig. 3f). Zygospores compressed ellipsoid, 100-107 × 125-144 × 68-90 μm; mesospore yellow-brown, smooth (Figs. 3g, h).

Distribution: Japan[S6] (Table 1).

Strain examined: nag101 (JPS009).

Remarks: Since Yamagishi[S18] described *Spirogyra minuticrassoidea* based on the field-collected material from Aichi prefecture, Japan, no further records of this species have previously been reported. The present Japanese material agrees with the species concept of *S. minuticrassoidea* by Yamagishi[S18] in having plane transverse walls, 6-7 chloroplasts, compressed ellipsoid zygospores and smooth mesospore. This species is morphologically similar to *S. crassoidea* Transeau and *S. ellipsospora* Transeau according to Transeau[S7]. However, this species differs from both species in having a smaller compressed ellipsoid zygospore and narrower vegetative cells.

Spirogyra mirabilis (Hassall) Kützing 1849: 438[S17]. (Figs. 2e-h)

Basionym: Zygnema mirabile Hassall 1843: 23[S19].

Species description: Vegetative cells 20-26 μm wide, 105-270 μm long; transverse walls plane; chloroplasts single per cell (Fig. 2e). Reproduction by aplanospores; sporangia inflated (Fig. 2f). Aplanospores ovoid, 23-32 × 41-53 μm; mesospore yellow-brown, smooth (Figs. 2g, h).

Distribution: Widely distributed in the United States, Europe, China and Japan[S6,S7,S9,S12-14] (Table S1).

Strain examined: shi0305 (JPS010).

Remarks: Transeau[S7] described morphological details of *Spirogyra mirabilis* such as its plane transverse walls, single chloroplasts, ovoid aplanospores and smooth mesospores. Transeau[S7] reported very rare scalariform conjugation in this species. We have not observed sexual reproduction in the present Japanese material under the present culture conditions.

Spirogyra chenii Jao 1935: 587[S8]. (Figs. 2i-m)

Species description: Vegetative cells 17-22 μm wide, 160-270 μm long; transverse walls plane; chloroplasts single per cell (Fig. 2i). Conjugation lateral and scalariform; conjugation

tubes formed by male gametangia; fertile female gametangia inflated on the both sides (Fig. 2k). Zygospores ellipsoid, 24-27 × 42-52 μm; mesospore yellow, smooth (Figs. 2l, m).
Distribution: China and Japan [S6,S9] (Table S1).
Strain examined: chiA307 (JPS011).
Remarks: The present cultured material agrees with the original description of *S. chenii* by Jao[S8] and Yamagishi[S6] in having plane transverse walls, single chloroplasts, conjugation tubes formed by male gametangia, female gametangia inflated on the both side and smooth single-layered mesospore wall.

Spirogyra pseudomaxima Kadłubowska 1979: 57[S20]. (Figs. 3i-n)
Species description: Vegetative cells 135-140 μm wide, 190-430 μm long; transverse walls plane; chloroplasts 7-8 per cell (Fig. 3i). Conjugation scalariform; conjugation tubes formed by both gametangia; fertile female gametangia cylindrical (Fig. 3j). Zygospores lenticular, 112-124 × 112-124 × 90-95 μm; mesospore two-layered, outer thin, with branched ribs; inner brown, reticulate (Figs. 3k-n).
Distribution: Denmark and Japan[S12] (Table S1).
Strain examined: chi0305 (JPS012).
Remarks. *Spirogyra pseudomaxima* was originally described by Kadłubowska[S20] based on the field-collected material from Denmark. The present Japanese material agrees with the species concept of *S. pseudomaxima* by Kadłubowska[S20] in having plane transverse walls, 5-7 chloroplasts, compressed ellipsoid zygospores and two-layered mesospore. This species has not previously been recorded from Japan[S6]. This species is similar to *S. maxim*a in having reticulate mesospore, but this species has a two-layered mesospore (Fig. 3n) whereas the mesospore of *S. maxima* is single-layered according to Transeau[S5] and Kadłubowska[S12].

Spirogyra punctata Cleve 1868: 23[S21]. (Figs. 4f-j)
Synonym: Temnogyra punctata (Cleve) Yamagishi 1963: 207[S11].
Species description: Vegetative cells 26-32 μm wide, 150-300 μm long; transverse walls plane; chloroplasts single per cell (Fig. 4f). Conjugation scalariform; conjugation tubes formed by both gametangia; fertile female gametangia cylindrical, sometimes slightly inflated (Fig. 4h). Gametangia adjoining sterile cells in both male and female filaments (Fig. 4g). Zygospores ellipsoid, 31-34 × 54-65 μm; mesospore yellow-brown, punctate (Figs. 4i, j).
Distribution: Widely distributed in Europe, America, Asia, Africa, and Australia[S6,S7,S9,S12-14] (Table S1).

Strain examined: Tpx8 (JPS013).
Remarks: Transeau[S7] described morphological details of this species such as having plane transverse walls, single chloroplasts, and punctate mesospores. The present Japanese material agrees with the species concept of *T. punctata* by Transeau[S7]. As *Spirogyra punctata,* this species was classified to the genus *Temnogyra* by Yamagishi[S11] because it has sterile cells adjoining gametangia. The present phylogenetic analysis either did not support the separation of this species from the genus *Spirogyra*.

Spirogyra semiornata Jao 1935: 604[S8]. (Figs. 1m-p)
Species description: Vegetative cells 26-32 μm wide, 250-380 μm long; transverse walls replicate; chloroplasts single per cell (Fig. 1m). Conjugation scalariform; conjugation tubes formed by both gametangia; fertile female gametangia cylindrical, sometimes slightly enlarged (Fig. 1n). Zygospores ellipsoid with obtuse ends, 35-42 × 78-107 μm; mesospore yellow-brown, smooth (Figs. 1o, p).
Distribution: Europe, Africa, China and Japan[S6,S7,S9,S12-14] (Table S1).
Strain examined: chiA304 (JPS014).
Remarks: *Spirogyra semiornata* was originally described by Jao[S8] based on the field-collected material from China. The present Japanese material agrees with the original description of *S. semiornata* by Jao[S8] in having replicate transverse walls, single chloroplasts and smooth mesospore. Although Jao[S8] reported both lateral and scalariform conjugation in this species, we observed only scalariform conjugation in Japanese material under the present culture conditions.

Spirogyra varians (Hassall) Kützing 1849: 439[S17]. (Figs. 2n-q)
Basionym: Zygnema varians Hassall 1843: 431[S19].
Species description: Vegetative cells 28-33 μm wide, 125-200 μm long; transverse walls plane; chloroplasts single per cell (Fig. 2n). Conjugation scalariform; conjugation tubes formed by male gametangia; fertile female gametangia inflated on the conjugation side (Fig. 2o). Zygospores ellipsoid, 30-34 × 50-53 μm; mesospore yellow-brown, smooth (Figs. 2p, q).
Distribution: Widely distributed in Europe, America, Asia, Africa, and Australia[S6,S7,S9,S12-14] (Table S1).
Strain examined: chi0102 (JPS015).
Remarks: Transeau[S7] described morphological details of this species such as having plane transverse walls, single chloroplasts, inflated female gametangia and smooth mesospores.

The present Japanese material agrees with the species concept of *S. varians* by Transeau[S7]. Transeau[S7] reported both lateral and scalariform conjugation in this species, we observed only scalariform conjugation of the Japanese material under the present culture conditions.

References for Supplementary Information

S1. Manhart, J. R. Phylogenetic analysis of green plant *rbc*L sequences. *Mol. Phylogenet. Evol*. **3**, 114–127 (1994).

S2. Sakayama, H., Nozaki, H., Kasaki, H. & Hara, Y. Taxonomic re-examination of *Nitella* (Charales, Charophyceae) from Japan, based on microscopic studies of oospore wall ornamentation and *rbc*L gene sequences. *Phycologia* **41**, 397–408 (2002).

S3. Shimada, A., Kanai, S. & Maruyama, T. Partial sequence of ribulose-1,5-bisphosphate carboxylase/oxygenase and the phylogeny of *Prochloron* and *Prochlorococcus* (Prochlorales). *J. Mol. Evol.* **40**, 671–677 (1995).

S4. Lemieux, C., Otis, C. & Turmel, M. Comparative Chloroplast Genome Analyses of Streptophyte Green Algae Uncover Major Structural Alterations in the Klebsormidiophyceae, Coleochaetophyceae and Zygnematophyceae. *Front. Plant Sci.* **7**, 697, doi:10.3389/fpls.2016.00697 (2016).

S5. Stancheva, R., Hall, J. D. & Sheath, R. G. Systematics of the genus *Zygnema* (Zygnematophyceae, Charophyta) from Californian watersheds. *J. Phycol.* **48**, 409–22 (2012).

S6. Yamagishi, T. *Illustrations of the Japanese fresh-water algae*. (ed. Hirose, H., Yamagishi, T.) 416–461 (Uchida Roukakuho Publishing Co., LTD., 1977).

S7. Transeau, E. N. *The Zygnemataceae*. (The Ohio State University Press, 1951).

S8. Jao, C. C. Studies on the freshwater Algae of China. I. Zygnemataceae from Szechwan. *Sinensia* **6**, 551-620 (1935).

S9. Jao, C. C. *Zygnemataceae. Flora Algarum Sinicarum Aquae Dulcis.* (Science Press, 1988).

S10. Transeau, E. N., Tiffany, L.H., Taft, C.E. & Li, L.C. New Species of Zygnemataceae. *Transactions of the American Microscopical Society* **53**, 208–230 (1934).

S11. Yamagishi, T. Classification of the Zygnemataceae. *Sci. Rep. Tokyo Kyoiku Daigaku B* **11**, 191-210 (1963).

S12. Kadłubowska, J. Z. *Conjugatophyceae I. Chlorophyta VIII. Zygnemales*. (ed. Ettl, H., Gerloff, J., Heynig, H. & Mollenhauer, D.) (G. Fischer, 1984).

S13. Rundina, L. A. *The Zygnematales of Russi*a *(Chlorophyta: Zygnematophyceae, Zygnematales).* (Nauka, 1998).

S14. Gauthier-Lièvre, L. Zygnèmacèes Africaines. *Nova Hedwigia* **20**, 1–210 (1965).

S15. Kützing, F. T. *Phycologia generalis oder Anatomie, Physiologie und Systemkunde der Tange. Mit 80 farbig gedruckten Tafeln, gezeichnet und gravirt vom Verfasser.* (F.A. Brockhaus, 1843).

S16. Vaucher, J. P. *Histoire des conferves d'eau douce. Contenant leurs différens modes de reproduction, et la description de leurs principales espèces, suivie de l'histoire des trémelles et des ulves d'eau douce*. (Chez J.J. Paschoud, 1803).

S17. Kützing, F. T. *Species algarum*. (F.A. Brockhaus, 1849).

S18. Yamagishi, T. New Species of the Zygnemataceae Collected in Japan. *Bot. Mag. Tokyo* **76**, 215-218 (1963).

S19. Hassall, A.H. Observations on some points in the anatomy and physiology of the freshwater algae. *Annals and Magazine of Natural History Series 1* **12**, 20-31 (1843).

S20. Kadłubowska, J. Z. & Christensen, T. Some Danish Zygnemataceae. *Bot. Tidsskr*. **74**, 165-173 (1979).

S21. Cleve, P.T. Försök till en monografi öfver de Svenska arterna af algfamiljen Zygnemaceae. *Nova Acta Regiae Societatis Scientiarum Upsaliensis series 3* **6**, 1-38 (1868).
